# Incidence and mortality trends of neglected tropical diseases and malaria in China and ASEAN countries from 1990 to 2019 and its association with the socio-demographic index

**DOI:** 10.1186/s41256-023-00306-1

**Published:** 2023-06-23

**Authors:** Qiao Liu, Wenxin Yan, Chenyuan Qin, Min Du, Yaping Wang, Min Liu, Jue Liu

**Affiliations:** 1 Department of Epidemiology and Biostatistics, School of Public Health, Peking University, No. 38, Xueyuan Road, Haidian District, Beijing, 100191 China; 2 Institute for Global Health and Development, Peking University, No. 5 Yiheyuan Road, Haidian, Beijing, 100871 China; 3 Global Center for Infectious Disease and Policy Research & Global Health and Infectious Diseases Group, Peking University, No. 38, Xueyuan Road, Haidian District, Beijing, 100191 China; 4 Key Laboratory of Epidemiology of Major Diseases (Peking University), Ministry of Education, No. 38, Xueyuan Road, Haidian District, Beijing, 100191 China

**Keywords:** Neglected tropical diseases, Malaria, China-ASEAN, Burden, Trends

## Abstract

**Background:**

People in China and the countries in the Association of Southeast Asian Nations (ASEAN) are affected by neglected tropical diseases and malaria (NTDM). In this study, we aimed to assess the current status and trends of NTDM burden from 1990 to 2019 in China and ASEAN countries, and also explore the association of NTDM burden with socio-demographic index (SDI).

**Methods:**

The data from the Global Burden of Diseases Study 2019 (GBD 2019) results were used. Absolute incidence and death number, and age-standardized incidence and mortality rate (ASIR and ASMR) of NTDM in China and ASEAN were extracted. The estimated annual percentage change (EAPC) and join-point regression in the rates quantified the trends. Nonlinear regression (second order polynomial) was used to explore the association between SDI and ASRs.

**Results:**

The ASIR of NTDM increased in China, Philippines, Singapore and Brunei, at a speed of an average 4.15% (95% CI 3.83–4.47%), 2.15% (1.68–2.63%), 1.03% (0.63–1.43%), and 0.88% (0.60–1.17%) per year. Uptrends of ASIR of NTDM in recent years were found in China (2014–2017, APC = 10.4%), Laos (2005–2013, APC = 3.9%), Malaysia (2010–2015, APC = 4.3%), Philippines (2015–2019, APC = 4.2%), Thailand (2015–2019, APC = 2.4%), and Vietnam (2014–2017, APC = 3.2%, all *P* < 0.05). Children < 5 had relatively low incidences but unexpectedly high mortality rates of NTDM in most ASEAN countries. Both incidence and mortality rates of NTDM were higher in older people. ASIR and ASMR of NTDM had a U-shaped association with SDI.

**Conclusions:**

The burden of NTDM in China and ASEAN countries was still huge and affects vulnerable and impoverished populations’ livelihoods, including children under the age of 5 and people aged 60 and older. Facing with the large burden and complex situation of NTDM in China and ASEAN countries, regional cooperating strategies are needed to reduce the burden of NTDM, so as to achieve the goal of elimination in the world.

**Supplementary Information:**

The online version contains supplementary material available at 10.1186/s41256-023-00306-1.

## Introduction

The countries in the Association of Southeast Asian Nations (ASEAN), including Brunei, Cambodia, Indonesia, Laos, Malaysia, Myanmar, Philippines, Singapore, Thailand, and Vietnam, are economically and socially integrated. The coronavirus disease 2019 (COVID-19) pandemic has brought the need for regional collaboration on prevention and control of infectious diseases to the fore, and ASEAN countries have resolved to address cross-country management of infectious diseases in late 2020 [1]. However, with different types of political intuitions, different religions, various levels of economic development, and, the most important, different types of health systems, there remains challenges for ASEAN countries to achieve regional coordination for the prevention and control of infectious diseases [2]. China-ASEAN dialogue relations have been established over 30 years [3]. As the two largest economies in the Asia Pacific region, China and ASEAN could be well positioned to exert a significant influence on Asian and even global health.

Neglected tropical diseases mostly affect people living in extreme poverty, and almost 30% of the population in ASEAN countries were living in extreme poverty [4]. Previous study had shown great burden of neglected tropical diseases and malaria (NTDM), in China and certain ASEAN countries. An alarming 5.2 million dengue cases were reported in 2019, and Asia contributes 70% of the global dengue burden [5]. Additionally, dengue represents a major economic threat, according to previous study estimating that almost US$1 billion in annual economic losses for ASEAN countries [6]. The age-standardized incidence rate of malaria in Southeast Asia was 134.47 per 100,000 in 2019, higher than most other regions outside of the African regions [7]. As reported by World Malaria Report 2022, South-East Asia Region had nine malaria endemic countries in 2021, accounting for 5.4 million cases and contributing 2% of the burden of malaria cases globally. [8] Rabies, with high case fatality rate, also represents an important public health threat in ASEAN countries. Although rabies has been declared free in Singapore and Malaysia, rabies outbreaks occurred at few states in recent years [9, 10]. Meanwhile, the remainder of ASEAN countries and China, especially the poorest areas of countries such as Indonesia or the Philippines, continue to report rabies cases. [11] It was also shown that Southeast Asia and several other tropical regions were experiencing increasing trends in the incidence of neglected tropical diseases [12].

The Sustainable Development Goals (SDGs) aimed to end the epidemics of NTDM by 2030. [13] Despite the decrease in mortality and morbidity from major infectious diseases, the world is not on track to meet the 2020 milestones of SGDs of certain infectious diseases, such as malaria and Dengue [14–16]. To achieve the goals of SDGs, huge efforts need to be taken by countries all over the world, especially for China and ASEAN countries, the two largest economies in the Asia Pacific region. However, a comprehensive description of NTDM in China and ASEAN and their position compared with the global level is lacking and emergingly needed.

Therefore, in this study, we aimed to assess the current status and trends of NTDM burden from 1990 to 2019 in China and ASEAN countries, using the data from Global Burden of Disease Study 2019 (GBD 2019) results, which consisted of a systematic and scientific effort to quantify the comparative magnitude of health losses due to diseases by sex, age, and location over time [17]. Moreover, we also aimed to explore the association of NTDM burden in China and ASEAN countries with the development status of countries, using the socio-demographic index (SDI) of each country. The SDI is a composite indicator of development status strongly correlated with health outcomes. It is the geometric mean of 0 to 1 indices of total fertility rate under the age of 25, mean education for those ages 15 and older, and lag distributed income per capita. As a composite, a country with an SDI of 0 would have a theoretical minimum level of development relevant to health, while a country with an SDI of 1 would have a theoretical maximum level [18]. Our study can provide a comprehensive perspective for better understanding the long-term trends and national differences in incidence and mortality from NTDM among China and ASEAN countries, which could help make global strategies to eliminate NTDM more scientific and sounder.

## Methods

### Study design

This is an observational study using data obtained from the GBD 2019 result tools. Annual number of incident number, incidences, death number, and mortality rates of neglected tropical diseases and malaria (NTDM) were extracted, by chosen specified diseases (malaria, cystic echinococcosis, dengue, and rabies) and the overall category. The socio-demographic index (SDI) of China and ASEAN countries from 1990 to 2019 was also extracted from the GBD 2019 result tools.

### Data collection and processing

Data were obtained from the GBD 2019 result tools, established by the GBD group [19]. The general methodological approaches to estimate the mortality were described elsewhere. [20] Briefly, all available data on causes of death or incidence were standardized and pooled into a single database used to generate cause-specific estimates by age, sex, year, and geography; then multiple models, such as cause of death ensemble modelling, disease model-Bayesian meta-regression, comorbidity correction and so on were used to estimate comparable data of different diseases across the world. [20]

We reported the incidence and death results of NTDM in China, ASEAN countries (Brunei, Cambodia, Indonesia, Laos, Malaysia, Myanmar, Philippines, Singapore, Thailand, and Vietnam) and the global total data from 1990 to 2019, and arranged incidence and death data into successive 5-year age intervals from < 5 years to 65–69 years, plus the 70 + years group.

### Statistical analysis

Absolute incidence and death number represented the actual situation of NTDM in each country, and its relative change was defined as $\frac{{Number}_{2019}-{Number}_{1990}}{{Number}_{1990}}\times 100\%$, which showed the overall change between 1990 and 2019. Age-standardized incidence rate (ASIR) and age-standardized mortality rate (ASMR), which were directly extracted from the GBD result tool, [19] were calculated by applying the age-specific rates to a GBD World Standard Population, and were used to compare populations with different age structures or for the same population over time in which the age profiles change accordingly.

Estimated annual percentage change (EAPC) was widely used to quantified the rate trend over a specific interval. A regression line was fitted to the natural logarithm of the rates (*y* = *α* + *βx* + *ε*, where *y* = ln(rate) and *x* = calendar year). EAPC was calculated as 100 × (*e*^*β*^-1), with 95% confidence intervals (CIs) obtained from the linear regression model. In this study, overall EAPC was calculated by the annual ASIR and ASMR of each category of infectious diseases in China and ASEAN countries, and EAPC in different age groups was calculated by the age-specific incidence and mortality rate. The term “increase” was used to describe trends when the EAPC and its lower boundary of 95% CI were both > 0. In contrast, “decrease” was used when the EAPC and its upper boundary of 95% CI, were both < 0. Otherwise, the term “stable” was used.

Join-point regression model was conducted to evaluate the temporal trend in the ASIRs and ASMRs. The best-fitting points were identified by the slope of changing trend and connected a set of statistically linear models on a logarithmic scale, and the number of join points were determined with maximum of 5. The join-point regression model was listed as follow: $E\left[y|x\right]={e}^{{\beta }_{0}+{\beta }_{1}x+{{\delta }_{1}\left(x-{\tau }_{1}\right)}^{+}+\dots +{{\delta }_{k}\left(x-{\tau }_{k}\right)}^{+}}$, where *k* denotes the number of turning points, *τ*_*k*_ denotes the unknown turning points, *β*_*0*_ denotes the invariant parameter, *β*_*1*_ denotes the regression coefficient, *δ*_*k*_ denotes the regression coefficient of the piecewise function of *k*. Annual percentage change (APC) and corresponding 95% CIs were obtained to quantify the piecewise trends of ASIR and ASMR.

Lastly, nonlinear regression (second order polynomial) was conducted to explore the association between SDI and ASRs in China and ASEAN countries throughout 1990 to 2019. A regression curve was fitted to the ASIR or ASMR ($y=\alpha +\beta x+\gamma {x}^{2}$, where *y* = the value of ASRs and *x* = SDI).

All the statistical analyses were performed by the R program (version 4.4.1).

## Results

### Burden and temporal trends of NTDM in China and ASEAN countries from 1990 to 2019

The incidence and death number of NTDM in China and ASEAN together accounted for 4.40% and 4.12% of global cases in 1990, and 4.99% and 2.43% in 2019. In 2019, the largest incident number of NTDM was from China (6.11 million), followed by Indonesia (3.42 million) and Philippines (1.85 million). However, the largest death number of NTDM was from Indonesia (11,029), followed by Philippines (2870) and China (1860). (Table 1).

**Table 1:** EAPCs and changes between 1990 and 2019 of NTDM burden in China and ASEAN countries

	Number			Age-standardized rate (per 100,000)		
	1990	2019	Change (%)	1990	2019	EAPC (%)
*Incidence*						
Global	276,737,307 (223,626,967 to 339,311,683)	289,730,013 (230,991,844 to 364,945,572)	4.69	4703.20 (3793.02 to 5747.54)	4006.85 (3186.96 to 5066.98)	− 0.50 (− 0.69 to − 0.30)
China	2,008,500 (1,569,540 to 2,628,858)	6,105,863 (2,161,046 to 11,576,293)	204.00	169.22 (133.54 to 220.74)	467.85 (162.45 to 902.32)	4.15 (3.83 to 4.47)
Brunei	2020 (983 to 3608)	3829 (2403 to 5941)	89.55	792.37 (401.94 to 1405.98)	861.32 (507.81 to 1392.00)	0.88 (0.60 to 1.17)
Cambodia	527,851 (442,607 to 623,532)	240,211 (179,665 to 332,021)	− 54.49	5420.95 (4589.69 to 6335.74)	1447.29 (1083.19 to 2012.58)	− 4.56 (− 5.08 to − 4.04)
Indonesia	4,382,681 (1,682,096 to 12,045,425)	3,419,779 (2,620,343 to 4,897,670)	− 21.97	2253.84 (878.06 to 6227.24)	1324.06 (1019.94 to 1890.96)	− 1.09 (− 1.72 to − 0.46)
Laos	105,264 (81,054 to 132,709)	112,414 (67,834 to 176,760)	6.79	2764.54 (2219.36 to 3384.51)	1574.07 (952.71 to 2441.21)	− 3.56 (− 4.68 to − 2.43)
Malaysia	330,542 (271,195 to 414,509)	463,484 (282,207 to 682,672)	40.22	1925.07 (1594.00 to 2401.49)	1486.64 (908.38 to 2173.06)	− 0.94 (− 1.50 to − 0.38)
Myanmar	1,536,594 (1,077,533 to 2,068,355)	456,233 (199,602 to 874,747)	− 70.31	3861.12 (2737.41 to 5148.00)	839.71 (371.38 to 1600.85)	− 2.37 (− 3.85 to − 0.87)
Philippines	648,380 (311,703 to 994,489)	1,845,524 (1,174,212 to 2,988,192)	184.64	1023.89 (499.38 to 1561.97)	1631.52 (1053.04 to 2585.68)	2.15 (1.68 to 2.63)
Singapore	28,730 (19,513 to 43,443)	58,231 (43,932 to 79,532)	102.68	928.35 (615.94 to 1441.38)	1013.91 (738.41 to 1448.57)	1.03 (0.63 to 1.43)
Thailand	1,642,924 (1,333,175 to 1,962,239)	706,517 (514,931 to 1,015,841)	− 57.00	2877.21 (2343.29 to 3401.04)	1080.62 (821.74 to 1469.34)	− 2.84 (− 3.48 to − 2.20)
Viet Nam	968,645 (718,588 to 1,246,127)	1,056,806 (827,134 to 1,351,504)	9.10	1426.03 (1060.70 to 1824.17)	1108.05 (858.25 to 1425.58)	− 1.46 (− 1.75 to − 1.18)
*Deaths*						
Global	1,034,119 (638,521 to 1,551,484)	747,344 (406,562 to 1,247,138)	− 27.73	18.06 (11.26 to 27.02)	10.32 (5.61 to 17.27)	− 1.89 (− 2.28 to − 1.49)
China	6677 (4369 to 23,511)	1860 (1307 to 2224)	− 72.14	0.67 (0.46 to 2.10)	0.11 (0.08 to 0.13)	− 5.77 (− 6.42 to − 5.11)
Brunei	1 (0 to 1)	1 (1 to 2)	101.48	0.45 (0.25 to 0.63)	0.41 (0.21 to 0.54)	− 0.08 (− 0.26 to 0.09)
Cambodia	965 (466 to 1924)	193 (108 to 308)	− 80.05	8.96 (4.50 to 17.21)	1.29 (0.77 to 1.99)	− 6.35 (− 7.70 to − 4.98)
Indonesia	20,736 (6184 to 37,411)	11,029 (4018 to 14,447)	− 46.81	10.62 (3.50 to 20.02)	5.13 (1.81 to 6.67)	− 2.20 (− 2.54 to − 1.85)
Laos	231 (116 to 379)	76 (40 to 126)	− 66.97	5.77 (3.15 to 9.36)	1.24 (0.69 to 2.01)	− 5.62 (− 7.03 to − 4.18)
Malaysia	331 (193 to 569)	283 (147 to 467)	− 14.40	2.05 (1.26 to 3.52)	0.95 (0.51 to 1.60)	− 3.18 (− 4.00 to − 2.36)
Myanmar	7702 (3815 to 15,985)	1215 (534 to 2796)	− 84.23	17.64 (8.73 to 36.84)	2.32 (1.08 to 5.34)	− 3.43 (− 6.10 to − 0.68)
Philippines	2931 (1792 to 4287)	2870 (1655 to 3430)	− 2.07	4.66 (3.18 to 7.13)	2.60 (1.65 to 3.10)	− 1.98 (− 2.37 to − 1.59)
Singapore	1 (1 to 2)	5 (2 to 8)	271.93	0.06 (0.03 to 0.07)	0.07 (0.03 to 0.11)	1.22 (− 0.19 to 2.65)
Thailand	1914 (1010 to 3389)	279 (139 to 398)	− 85.40	3.46 (1.85 to 6.07)	0.43 (0.20 to 0.61)	− 6.33 (− 6.82 to − 5.84)
Viet Nam	1089 (516 to 2680)	343 (222 to 559)	− 68.45	1.83 (0.86 to 4.52)	0.40 (0.26 to 0.63)	− 6.11 (− 7.09 to − 5.12)

EAPC, Estimated annual percentage change; NTDM, Neglected tropical diseases and malaria; ASEAN, Association of South East Asian Nations

**Fig. 1:**
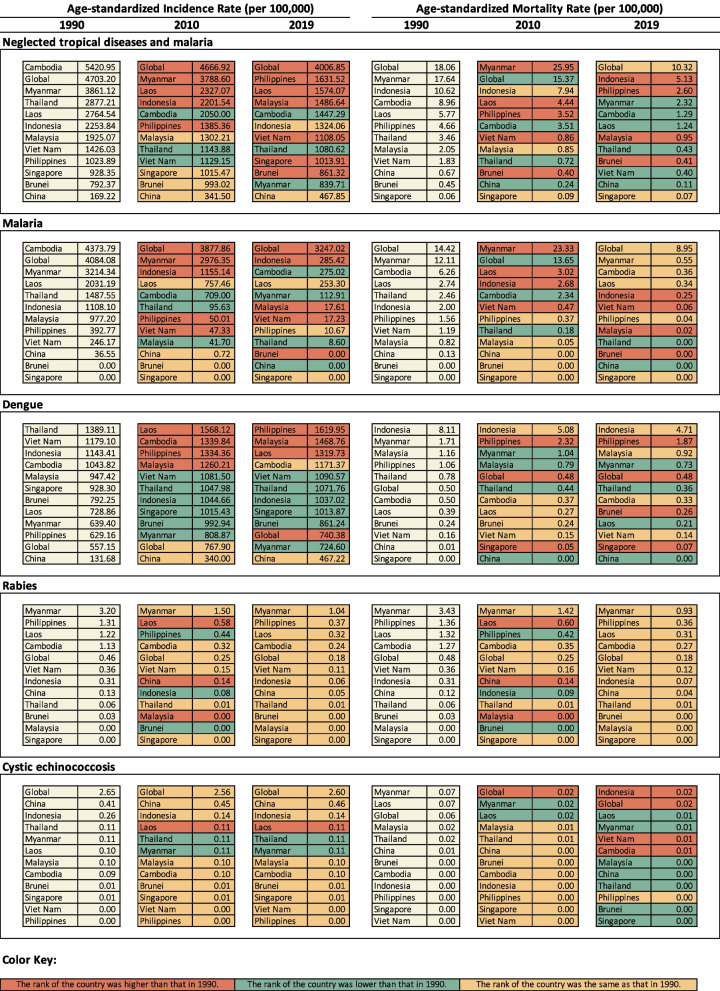
Age-standardized incidence and mortality rates of neglected tropical diseases and malaria, malaria, dengue, rabies, and cystic echinococcosis in China and ASEAN countries, in 1990, 2010 and 2019

**Fig. 2:**
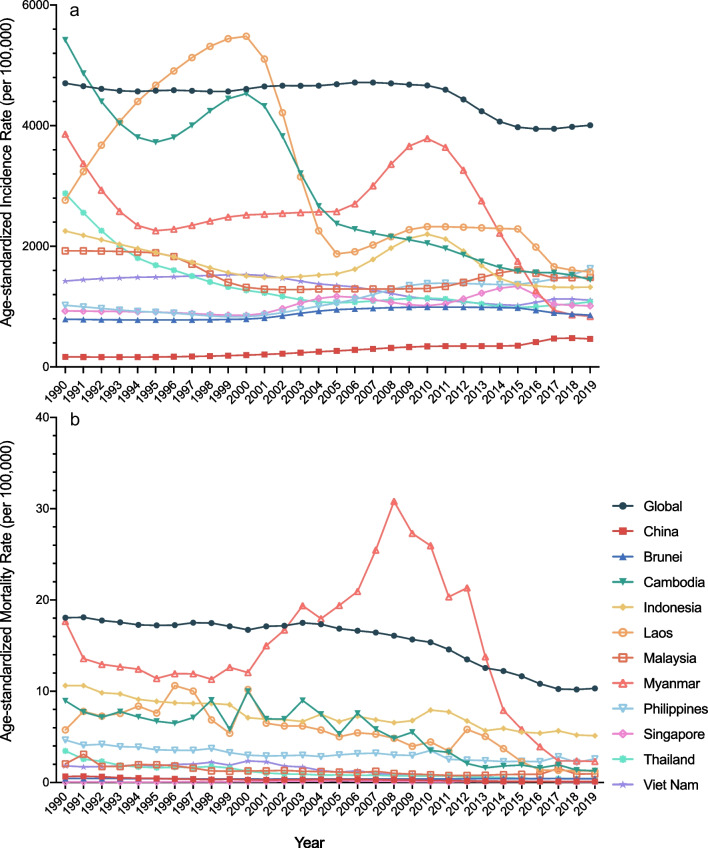
Age-standardized incidence and mortality rates of neglected tropical diseases and malaria in China and ASEAN countries, from 1990 to 2019. **a** Age-standardized incidence rates (per 100,000); **b** Age-standardized mortality rates (per 100,000)

The overall NTDM ASIRs and ASMRs in China and ASEAN were lower than the global level in 2019. However, only two countries (Myanmar and China) had lower dengue ASIRs than the global level, and four countries (Indonesia, Philippines, Malaysia and Myanmar) had higher dengue ASMRs than the global level. As for the rabies, Myanmar had the highest ASIR (1.04 per 100,000) and ASMR (0.93 per 100,000) in 2019, followed by Philippines (ASIR = 0.37 per 100,000; ASMR = 0.36 per 100,000) and Laos (ASIR = 0.32 per 100,000; ASMR = 0.31 per 100,000). Indonesia had the third-highest ASIR (0.14 per 100,000) and the highest ASMR (0.02 per 100,000) of cystic echinococcosis in 2019. (Figs. 1 and 2).

While the ASIR decreased from 1990 to 2019 at global level and in most ASEAN countries, the ASIR of NTDM increased in China, Philippines, Singapore and Brunei, at a speed of an average 4.15% (95% CI 3.83–4.47%), 2.15% (1.68–2.63%), 1.03% (0.63–1.43%), and 0.88% (0.60–1.17%) per year. The ASIR of Dengue increased in most countries with the speediest in China (EAPC = 4.83%, 95% CI 4.57–5.09%), followed by Philippines (EAPC = 3.71%, 95% CI 3.40–4.03%). Additionally, the ASMR of dengue in Philippines also increased at a speed of an average 2.42% (95% CI 1.89–2.95%) per year from 1990 to 2019. (Table 1, Additional file 1: Tables S1 and S2, and Additional file 1: Fig. S1).

Although the NTDM ASIRs and ASMRs in most countries had overall downtrends from 1990 to 2019, there were some periods in which the ASIR and ASMR showed uptrends, according to the join-point regression results. The global ASIR of NTDM showed an uptrend from 2015 to 2019 (APC = 0.4%, 95% CI 0.1–0.7%). Same uptrends in recent years were also found in China (from 2014 to 2017, APC = 10.4%, 95% CI 6.1–14.9%), Laos (from 2005 to 2013, APC = 3.9%, 95% CI 2.5–5.3%), Malaysia (from 2010 to 2015, APC = 4.3%, 95% CI 3.6–5.1%), Philippines (from 2015 to 2019, APC = 4.2%, 95% CI 3.7–4.8%), Thailand (from 2015 to 2019, APC = 2.4%, 95% CI 1.9–2.8%), and Vietnam (from 2014 to 2017, APC = 3.2%, 95% CI 1.5–4.9%). The ASMR of NTDM in China and ASEAN countries had not shown uptrends in recent years except Brunei, which showed an uptrend of NTDM ASMR from 2008 to 2016 (APC = 1.3%, 95% CI 1.0–1.7%). (Table 2) Additionally, ASIR and ASMR of dengue also showed uptrends in recent years in most countries: ASIR in China (APC = 10.4% from 2014 to 2017), Cambodia (APC = 5.1% from 2017 to 2019), Philippines (APC = 5.2% from 2016 to 2019), Thailand (APC = 2.4% from 2015 to 2019) and Vietnam (APC = 4.4% from 2014 to 2017); and ASMR of dengue in Cambodia (APC = 1.2% from 2010 to 2019), Laos (APC = 13.1% from 2016 to 2019), Singapore (APC = 4.2% from 2004 to 2019) and Vietnam (APC = 1.1% from 2001 to 2019). Notably, ASMR of dengue in Philippines had an uptrend throughout 1990 to 2019 (APC = 2.5%, 95% CI 2.0–3.1%). Moreover, ASIR of malaria in Laos (APC = 10.5% from 2011 to 2015) and Malaysia (APC = 28.0% from 2015 to 2019) also showed uptrends in recent years. (Additional file 1: Table S3).

**Table 2:** Join-point regression results, periods with uptrends of ASIRs and ASMR of NTDM

Location	Start year	End year	APC (95% CI) (%)	*P* value
*ASIR*				
Global	1998	2007	0.4 (0.3 to 0.5)	< 0.001
	2015	2019	0.4 (0.1 to 0.7)	0.026
China	1995	2000	3.7 (3.2 to 4.1)	< 0.001
	2000	2009	6.3 (6 to 6.6)	< 0.001
	2014	2017	10.4 (6.1 to 14.9)	< 0.001
Brunei	2000	2005	4 (3.5 to 4.6)	< 0.001
	2005	2010	0.8 (0.5 to 1.2)	< 0.001
Cambodia	1994	2001	2.7 (1.7 to 3.7)	< 0.001
Indonesia	2004	2010	8.3 (7.4 to 9.2)	< 0.001
Laos	1990	1993	13.6 (9.6 to 17.7)	< 0.001
	1993	1998	5.5 (3.9 to 7.2)	< 0.001
	2005	2013	3.9 (2.5 to 5.3)	< 0.001
Malaysia	2010	2015	4.3 (3.6 to 5.1)	< 0.001
Myanmar	1994	2006	1.4 (1 to 1.9)	< 0.001
	2006	2010	10.5 (8.1 to 13)	< 0.001
Philippines	2000	2006	5.5 (5.2 to 5.8)	< 0.001
	2006	2009	6.8 (6.1 to 7.4)	< 0.001
	2015	2019	4.2 (3.7 to 4.8)	< 0.001
Singapore	2000	2005	6.9 (4.4 to 9.4)	< 0.001
	2010	2014	7.5 (4.5 to 10.6)	< 0.001
Thailand	2005	2010	2 (1.5 to 2.4)	< 0.001
	2015	2019	2.4 (1.9 to 2.8)	< 0.001
Viet Nam	1990	2000	0.7 (0.6 to 0.8)	< 0.001
	2014	2017	3.2 (1.5 to 4.9)	0.001
*ASMR*				
China	2000	2005	3.7 (2.1 to 5.2)	< 0.001
Brunei	2001	2005	1.8 (0.5 to 3.2)	0.010
	2008	2016	1.3 (1 to 1.7)	< 0.001
Myanmar	1998	2008	10 (7.7 to 12.4)	< 0.001
Singapore	1998	2005	14.6 (6.3 to 23.5)	0.001

APC, annual percentage change; ASIR, age-standardized incidence rate; ASMR, age-standardized mortality rate; NTDM, neglected tropical diseases and malaria

### Sex and age differences of NTDM in China and ASEAN countries

**Fig. 3:**
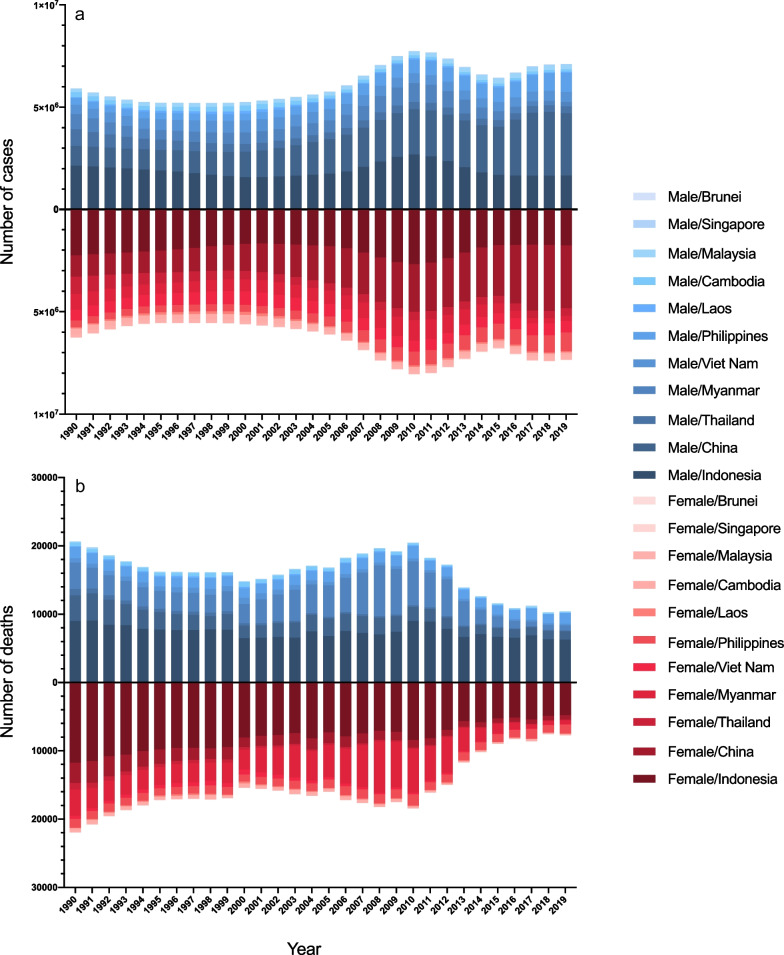
Incidence and death number of neglected tropical diseases and malaria in China and ASEAN countries, by sex, from 1990 to 2019. **a** number of cases; **b** number of deaths

As shown in Fig. 3, the incident number of NTDM was close in the male and the female from 1990 to 2019, in China and ASEAN countries. In 2019, there were a total of 7.36 million women and 7.11 million men infected by NTDM in China and ASEAN countries. The death number of women was larger than men in 1990 (21,939 women vs 20,638 men), yet smaller in 2019 (7708 women vs 10,447 men). The cases and deaths of rabies of women were much less than men throughout 1990 to 2019. (Additional file 1: Fig.S2).

**Fig. 4:**
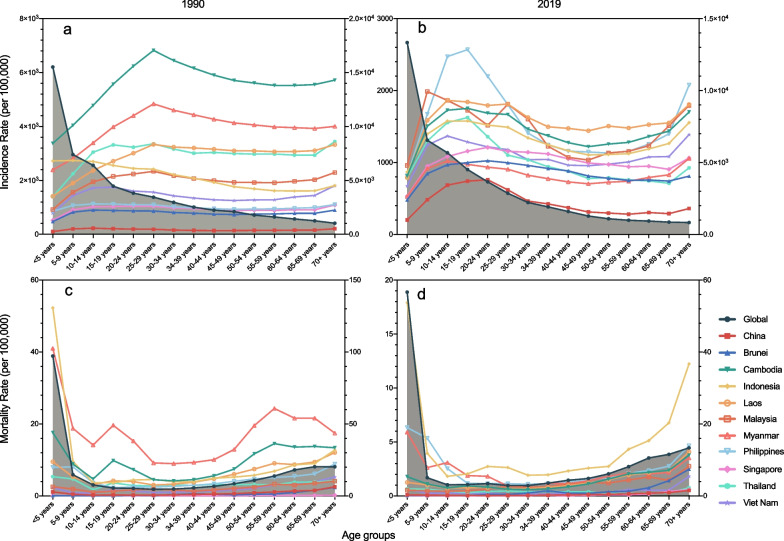
Age-specific incidence and mortality rates of neglected tropical diseases and malaria in China and ASEAN countries, in 1990 and 2019. The global line fits the right Y axis, and other lines fit the left axis. **a** Incidence (per 100,000) in 1990; **b** Incidence (per 1990) in 2019; **c** Mortality rate (per 100,000) in 1990; **d** Mortality rate (per 100,000) in 2019

The global incidences of NTDM decreased with age, however, the incidence of NTDM in China and ASEAN countries were higher in 25–29 years group in 1990 and 15–29 years groups in 2019, both increasing in the oldest age group. The age distribution of global NTDM mortality rates was the same as that in China and ASEAN countries: higher in the youngest and oldest age groups, in 1990 and 2019. Philippines had the highest incidences of NTDM in most age groups, and Indonesia had the highest mortality rates of NTDM in most age groups. (Fig. 4) In China and ASEAN countries, cystic echinococcosis showed lower incidences of younger and older people, yet higher mortality rates of younger and older people. The age distribution of dengue and malaria was similar to cystic echinococcosis, with dengue mortality rates of children under the age of 5 much higher than other age groups, and malaria mortality rates of people aged 15–20 also relatively higher. Incidence and mortality rates of rabies were much higher in adolescence aged 10–15 than other age groups. (Additional file 1: Fig. S3).

**Fig. 5:**
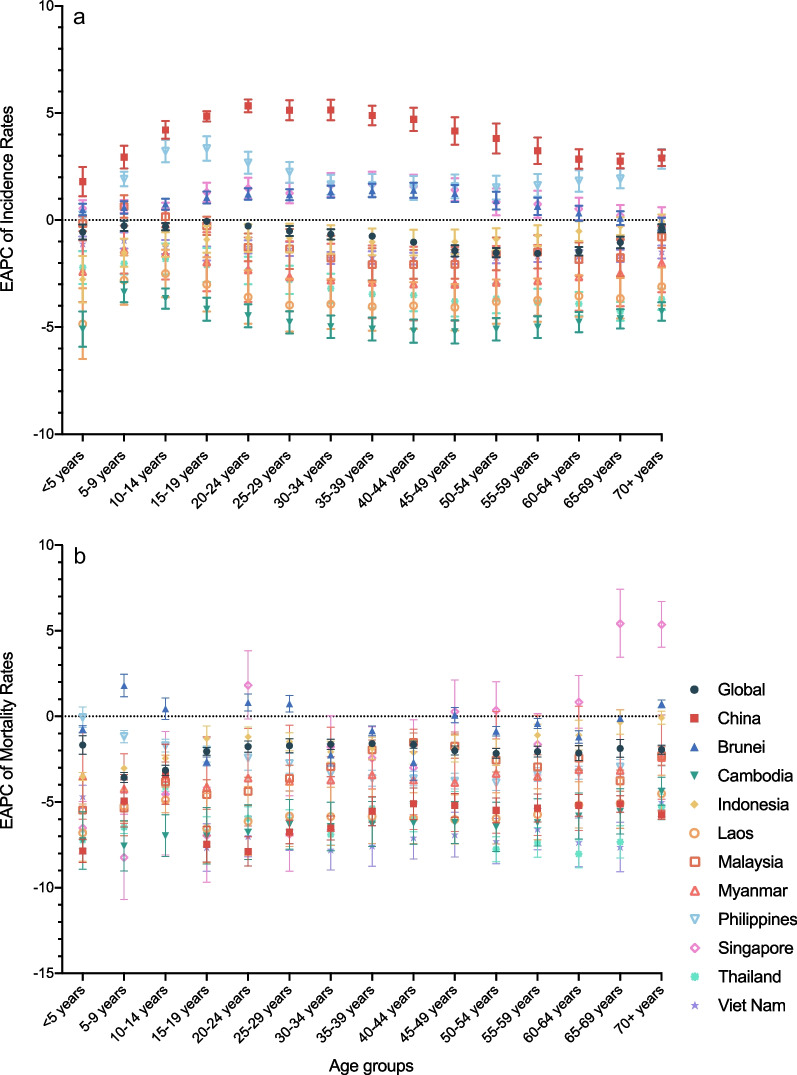
EAPC in different age groups of neglected tropical diseases and malaria in China and ASEAN countries, and the corresponding 95% Confident Intervals, from 1990 to 2019. **a** EAPC (%) of Incidences; **b** EAPC (%) of Mortality Rates. EAPC, estimated annual percentage change

All the age groups in China and Philippines had uptrends in incidences of NTDM from 1990 to 2019, with the speediest increase in 20–24 years group in China (EAPC = 5.34%, 95% CI 5.05–5.63%) and 15–19 years group in Philippines (EAPC = 3.34%, 95% CI 2.77–3.92%). Most age groups in Brunei and Singapore also showed uptrends in incidences of NTDM from 1990 to 2019, with the speediest increase in 40–44 years group (EAPC = 1.39%, 95% CI 1.05–1.74%) and 35–39 years group in Singapore (EAPC = 1.67%, 95% CI 1.09–2.26%). Nevertheless, only a few age groups in certain countries had uptrends of NTDM mortality rates, such as 65–69 years group (EAPC = 5.42%, 95% CI 3.45–7.42%) and 70 + years group (EAPC = 5.37%, 95% CI 4.05–6.70%) in Singapore, 5–9 years group (EAPC = 1.81%, 95% CI 1.15–2.46%), 20–24 years group (EAPC = 0.81%, 95% CI 0.31–1.31%), 25–29 years group (EAPC = 0.74%, 95% CI 0.25–1.23%) and 70 + years group (EAPC = 0.74%, 95% CI 0.51–0.96%) in Brunei. (Fig. 5) The incidences of cystic echinococcosis increased in most age groups in China, with the speediest in children under the age of 5 (EAPC = 1.46%, 95% CI 1.17–1.75%); the mortality rates of dengue increased much speedier in Singapore than in other countries, with the speediest in 70 + years group (EAPC = 24.11%, 95% CI 20.30–28.04%); and both incidence (EAPC = 6.35%, 95% CI 4.23–8.52%) and mortality (EAPC = 7.14%, 95% CI 3.94–10.43%) rates of rabies increased in children under the age of 5 in Singapore. (Additional file 1: Fig. S4).

### The association between SDI and ASRs of NTDM in China and ASEAN countries

**Fig. 6:**
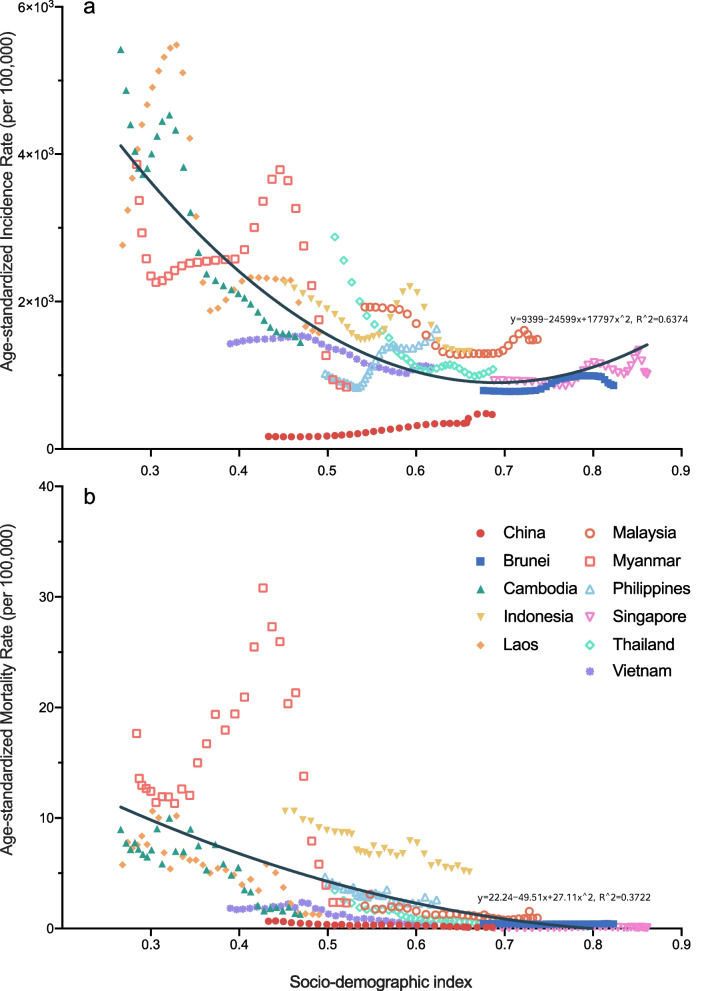
The correlation between age-standardized incidence and mortality rates of neglected tropical diseases and malaria and socio-demographic index in China and ASEAN countries. **a** association of Age-standardized incidence rate with socio-demographic index; **b** association of Age-standardized mortality rate with socio-demographic index

Overall, the ASIR and ASMR of NTDM had a U-shaped association with SDI in China and ASEAN countries, with the peak point appearing SDI around 0.69 for ASIR and 0.91 for ASMR. (Fig. 6) The ASMR of cystic echinococcosis also had a U-shaped association with SDI, with the peak point appearing SDI around 0.70. The ASIR and ASMR of malaria had a U-shaped association with SDI, with the peak point appearing SDI around 0.70 for ASIR and 0.78 for ASMR. The ASIR and ASMR of rabies also had a U-shaped association with SDI, with the peak point appearing SDI around 0.75 for ASIR and 0.74 for ASMR. (Additional file 1: Fig. S5).

## Discussion

To the best of our knowledge, this is the first comprehensive effort to describe the incidence and death of NTDM in China and ASEAN countries, also estimating their long-term trends throughout the past three decades, and accessing the association of the incidence and mortality rates of NTDM with the socio-demographic index. China, Indonesia and Philippines were countries with huge absolute incidence and death number of NTDM. Though the ASIRs and ASMRs of NTDM in China and ASEAN countries were lower than the global level, certain countries had higher ASIRs or ASMRs of dengue, rabies and cystic echinococcosis than the global level. ASIR of NTDM had an overall uptrend in China, Philippines, Singapore and Brunei from 1990 to 2019, while the NTDM ASIRs and ASMRs in most countries had overall downtrends from 1990 to 2019. However, there were some periods, especially recent years, in which the ASIR and ASMR showed uptrends, according to the join-point regression results. Sex disparity was not obvious in NTDM, but the incidence and death amount of rabies of women were much smaller than men throughout 1990 to 2019. The incidences of NTDM in China and ASEAN countries were higher in 25–29 years group in 1990 and 15–29 years group in 2019, both increasing in the oldest age groups; and the mortality rates were higher in both youngest and oldest groups. A U-shaped association of ASIR and ASMR of NTDM with SDI in China and ASEAN countries was also observed, indicating there were higher ASIR or ASMR in countries with the lowest and the highest SDI. Facing with the large burden and complex situation of NTDM in China and ASEAN countries, immediate and effective national cooperation are needed to reduce cases and deaths of NTDM and eliminate these diseases globally.

Our results, consistent with the findings of previous studies, [4, 16, 21–23] showed that the burden of dengue and rabies in China and ASEAN countries was above the world average level. Among China and ASEAN countries, Philippines and Indonesia had the highest ASIR (1619.95 per 100,000) and the highest ASMR (4.71 per 100,000) in 2019, respectively; and both ASIR and ASMR of rabies in Myanmar was the highest throughout 1990 to 2019. Meanwhile, the tremendous number of dengue cases in China also brought great concern. Furthermore, during the COVID-19 pandemic, the rate of dengue has continuously increased in ASEAN countries [21]. Climate change affects the survival and dispersion of *Aedes aegypti* and *Aedes albopictus* and transmission rates of viral pathogens of dengue [24], yet it was recommended to change vector control strategies during the epidemic wet seasons into a whole year to optimize the economic cost and burden of dengue and other arboviral diseases. [25] One of the effective ways to prevent the spread of dengue is the installation of water pipelines to residents in rural mountains to prevent mosquitoes from breeding in stored water [25, 26]. Besides, the dengue vaccine in Philippines, called Dengvaxia, which had a pooled efficacy amounting to 65.6% decrease in dengue with symptoms in people aged nine or older, could also serve as way of preventing dengue fever [27, 28]. Although Myanmar has made impressive progress against many infectious diseases, rabies was responsible for more deaths in Myanmar than malaria in recent years [22, 29]. In Myanmar, as well as in Cambodia, there was no national rabies program or coordinated canine vaccination campaign [23]. Therefore, appropriate fundings and integration of effective disease prevention strategies are necessary in Myanmar and other countries with rabies endemicity.

Despite the overall downtrends of NTDM burden from 1990 to 2019, certain diseases showed uptrends in recent years in China and ASEAN countries, according to the join-point regression results of our study. The increasing ASIR of NTDM in China (2014–2017), Malaysia (2010–2015), Philippines (2015–2019), Thailand (2015–2019), and Vietnam (2014–2017) addressed importance of taking more multifaceted and multisectoral actions to reduce the burden of NTDM in China and ASEAN countries to meet the global elimination goal [13]. One of the principles that WHO’s 2030 NTD elimination road map adopted is ‘leave no one behind’, which means taking explicit steps to end extreme poverty, curb inequalities, confront discrimination and fast track progress for the furthest behind [30]. Since almost 30% of the population in ASEAN countries were living in extreme poverty, the ‘leave no one behind’ principle could help reverse the uptrends of NTDM burden in China and ASEAN countries.

Our results showed that children under the age of five had relatively low incidences but unexpectedly high mortality rates of NTDM in most ASEAN countries, especially in Indonesia, Philippines and Myanmar. Additionally, both incidence and mortality rates of NTDM were higher in older people. On the one hand, NTDM affects children’s livelihoods in ASEAN countries. Helminthic infections with *Trichuris trichiura* or *Ascaris lumbricoides* were statistically associated with anemia and iron deficiency anemia, leading to incidences of low-birth-weight infants and inadequate growth and mental development in children, as well as high maternal mortality and low productivity in adults [31]. In Philippines in 2013, about 500,000 school-aged children required preventive chemotherapy for schistosomiasis [32]. Enterovirus 71 caused meningoencephalitis and Hand-Foot-and-Mouth disease in children, which could lead to fatal cases [33]. On the other hand, with decreased immunity and other underlying diseases, the prevention strategies of NTDM for elderly population were also in urgent needs.

This study also observed a U-shaped association of ASIR and ASMR of NTDM with SDI in China and ASEAN countries, with the peak point appearing SDI around 0.69 for ASIR and 0.91 for ASMR. The ASIR became higher when the SDI was over 0.69 might be because countries with the highest SDI, including Singapore and Brunei, had increased ASIR of NTDM over time. Unchecked urbanization without sanitation could promote the emergence of urban helminth infections [34]. It was reported that China, Malaysia and Singapore had mostly failed to shift their attention towards new innovations for the neglected tropical diseases, including new drugs, diagnostics, vaccines and vector control. This fact might lead these countries beset by widespread antimicrobial resistance, emerging arbovirus infections, and urban helminth infections [35]. Besides, political instability, conflict and human migrations in Myanmar and in and around the South China Sea could also make China and ASEAN countries important reservoirs for NTDM [36, 37]. Therefore, researchers have to look to the development of new or improved technologies for the prevention of vulnerable and impoverished populations.

Our study had some limitations. First, we described the overall situation of NTDM in China and ASEAN countries, but only four individual diseases were reported, due to the statistical characteristics of GBD results. Additionally, some important regional diseases such as melioidosis, chikungunya, and Japanese encephalitis have no estimation in the GBD [35]. Second, data from GBD results was estimated by several models, and the real burden of NTDM could be underestimated [20]. Nevertheless, our findings could alert the world that it was of vital importance to take regional cooperation to control NTDM, especially during the COVID-19 pandemic.

## Conclusions

The burden of NTDM in China and ASEAN countries was still huge and affects vulnerable and impoverished populations’ livelihoods, including children under the age of 5 and people aged 60 and older. There was an urgent need of actions to reverse the uptrends of incidence and mortality rates of NTDM, including dengue, malaria, rabies and cystic echinococcosis in recent years, in certain countries. Despite the difficulties, immediate and effective actions need to be taken to make China and ASEAN countries—which have different types of political intuitions, different religions, various levels of economic development, and different types of health systems—establish a regional cooperating strategy to reduce the burden of NTDM, so as to achieve the goal of elimination of these diseases in the world.

## Supplementary Information

**Additional file 1**. Supplementary tables and figures of *Incidence and mortality trends of neglected tropical diseases and malaria in China and ASEAN countries from 1990 to 2019 and its association with the Socio-demographic Index*.

## Data Availability

Data are available from the corresponding author by request.
