# Supplementary material for: Incidence and mortality trends of neglected tropical diseases and malaria in China and ASEAN countries from 1990 to 2019 and its association with the socio-demographic index
Source: Glob Health Res Policy. 2023 Jun 23;8:22. doi: 10.1186/s41256-023-00306-1 (PMC10288805; doi:10.1186/s41256-023-00306-1)
Supplement: Supplementary file 1 — Additional file 1. Supplementary tables and figures of Incidence and mortality trends of neglected tropical diseases and malaria in China and ASEAN countries from 1990 to 2019 and its association with the Socio-demographic Index. [file 41256_2023_306_MOESM1_ESM.pdf]

Incidence and mortality trends of neglected tropical diseases and malaria in China and ASEAN countries from 1990 to 2019 and its association with the Socio-demographic Index

Supplementary Files

Contents

Supplementary table 1. EAPCs and changes between 1990 and 2019 of cystic echinococcosis, dengue, malaria and rabies incidence in china and asean countries. 3

Supplementary table 2. EAPCs and changes between 1990 and 2019 of cystic echinococcosis, dengue, malaria and rabies mortality in china and asean countries. 4

Supplementary table 3. join-point regression results, periods with uptrends of asirs and asmr of cystic echinococcosis, dengue, malaria and rabies. 4

Supplementary figure 1. age-standardized incidence and mortality rates of cystic echinococcosis, dengue, malaria, and rabies in china and asean countries, from 1990 to 2019. 9

Supplementary figure 2. incident and death number of cystic echinococcosis, dengue, malaria, and rabies in china and asean countries, by sex, from 1990 to 2019. 13

supplementary figure 3. age-specific incidence and mortality rates of cystic echinococcosis, dengue, malaria, and rabies in china and asean countries, in 1990 and 2019. 17

Supplementary figure 4. EAPC in different age groups of cystic echinococcosis, dengue, malaria, and rabies in china and asean countries, and the corresponding 95% confident intervals, from 1990 to 2019. 21

supplementary figure 5. the correlation between age-standardized incidence and

mortality rates of cystic echinococcosis, dengue, malaria, and rabies and socio-demographic index in china and asean countries. **25**

Supplementary Table 1. EAPCs and changes between 1990 and 2019 of Cystic echinococcosis, Dengue, Malaria and Rabies incidence in China and ASEAN countries.

		Number			Age-standardized Rate (per 100,000)		
		1990	2019	Change (%)	1990	2019	EAPC (%)
**Cystic echinococcosis**	Global	134980 (93141 to 195144)	207368 (137807 to 303233)	53.63	2.65 (1.87 to 3.7)	2.6 (1.72 to 3.79)	-0.18 (-0.24 to -0.12)
	China	4975 (1540 to 11580)	6665 (2962 to 13327)	33.97	0.41 (0.14 to 0.89)	0.46 (0.19 to 0.95)	0.57 (0.49 to 0.65)
	Brunei	0 (0 to 0)	0 (0 to 0)	76.23	0.01 (0 to 0.01)	0.01 (0 to 0.01)	0.01 (0.01 to 0.01)
	Cambodia	10 (2 to 29)	17 (4 to 49)	77.42	0.09 (0.03 to 0.24)	0.1 (0.03 to 0.28)	0.11 (0.06 to 0.17)
	Indonesia	499 (119 to 1524)	354 (97 to 957)	-29.08	0.26 (0.07 to 0.74)	0.14 (0.04 to 0.36)	-3.23 (-3.85 to -2.62)
	Laos	4 (1 to 14)	8 (2 to 23)	78.64	0.1 (0.03 to 0.28)	0.11 (0.03 to 0.31)	0.03 (0.02 to 0.05)
	Malaysia	18 (5 to 54)	32 (9 to 86)	77.35	0.1 (0.03 to 0.26)	0.1 (0.03 to 0.27)	0.01 (0.01 to 0.01)
	Myanmar	44 (11 to 133)	59 (15 to 167)	33.45	0.11 (0.03 to 0.28)	0.11 (0.03 to 0.3)	0.02 (0.01 to 0.03)
	Philippines	2 (1 to 3)	3 (1 to 5)	70.94	0 (0 to 0)	0 (0 to 0)	0 (0 to 0)
	Singapore	0 (0 to 0)	0 (0 to 1)	90.43	0.01 (0 to 0.01)	0.01 (0 to 0.01)	0.01 (0 to 0.01)
	Thailand	65 (16 to 201)	73 (23 to 186)	12.79	0.11 (0.03 to 0.31)	0.11 (0.03 to 0.3)	-0.01 (-0.02 to 0)
	Viet Nam	2 (1 to 3)	3 (1 to 4)	36.29	0 (0 to 0)	0 (0 to 0)	0.25 (0.2 to 0.29)
**Dengue**	Global	30667501 (13221732 to 67065585)	56878730 (37083098 to 101350556)	85.47	557.15 (243.32 to 1212.53)	740.38 (478.25 to 1323.07)	1.2 (1.02 to 1.37)
	China	1558423 (1189692 to 1916419)	6096826 (2151664 to 11566646)	291.22	131.68 (101.2 to 161.49)	467.22 (161.82 to 901.62)	4.83 (4.57 to 5.09)
	Brunei	2020 (983 to 3608)	3829 (2403 to 5941)	89.55	792.25 (401.83 to 1405.85)	861.24 (507.74 to 1391.93)	0.88 (0.6 to 1.17)
	Cambodia	107133 (73861 to 160987)	194685 (147096 to 263079)	81.72	1043.82 (758.14 to 1551.06)	1171.37 (887.8 to 1594.67)	0.76 (0.32 to 1.2)
	Indonesia	2173181 (618623 to 9995522)	2655180 (2243011 to 3450631)	22.18	1143.41 (328.46 to 5182.99)	1037.02 (881.07 to 1342.97)	0.06 (-0.12 to 0.25)
	Laos	28388 (12698 to 49709)	94474 (56763 to 140589)	232.8	728.86 (392.69 to 1193.72)	1319.73 (796.44 to 1933.54)	1.52 (0.96 to 2.09)
	Malaysia	167407 (111591 to 252176)	457684 (277435 to 675961)	173.4	947.42 (648.65 to 1425.78)	1468.76 (894.29 to 2150.69)	1.9 (1.73 to 2.08)
	Myanmar	263713 (87016 to 512501)	392554 (192026 to 669276)	48.86	639.4 (215.06 to 1243.97)	724.6 (358.65 to 1233.58)	1.34 (1.05 to 1.62)
	Philippines	392057 (79370 to 730090)	1832712 (1165380 to 2979228)	367.46	629.16 (148.95 to 1147.73)	1619.95 (1044.81 to 2574.39)	3.71 (3.4 to 4.03)
	Singapore	28729 (19512 to 43441)	58228 (43929 to 79529)	102.69	928.3 (615.9 to 1441.33)	1013.87 (738.37 to 1448.53)	1.01 (0.61 to 1.42)
	Thailand	798026 (499380 to 1113112)	699668 (508746 to 1009397)	-12.33	1389.11 (875.56 to 1906.71)	1071.76 (817.13 to 1461)	-0.9 (-1.29 to -0.51)
	Viet Nam	812956 (598470 to 1051771)	1038968 (812231 to 1333875)	27.8	1179.1 (870.85 to 1500.71)	1090.57 (843.72 to 1408.31)	-0.61 (-0.84 to -0.38)
**Malaria**	Global	242785690 (200766324 to 298545031)	231357372 (186034444 to 290217185)	-4.71	4084.08 (3388.02 to 4998.95)	3247.02 (2602.06 to 4109.56)	-0.8 (-1.02 to -0.58)
	China	438309 (304952 to 1041798)	0 (0 to 0)	-100	36.55 (25.56 to 86.58)	0 (0 to 0)	-42.75 (-52.04 to -31.66)
	Brunei	0 (0 to 0)	0 (0 to 0)	–	0 (0 to 0)	0 (0 to 0)	–
	Cambodia	420439 (348117 to 505594)	45389 (13807 to 109688)	-89.2	4373.79 (3663.27 to 5196.53)	275.02 (83.68 to 664.48)	-9.02 (-10.05 to -7.98)
	Indonesia	2206036 (841912 to 5122997)	760372 (245048 to 1910905)	-65.53	1108.1 (436.5 to 2515.72)	285.42 (90.91 to 724.71)	-3.27 (-4.77 to -1.75)
	Laos	76714 (63941 to 93075)	17872 (3666 to 63408)	-76.7	2031.19 (1702.71 to 2447.3)	253.3 (52 to 898.34)	-8.34 (-10.25 to -6.39)
	Malaysia	163070 (141783 to 184743)	5717 (2317 to 12398)	-96.49	977.2 (850.23 to 1106.21)	17.61 (7.14 to 38.2)	-15.56 (-16.8 to -14.31)
	Myanmar	1270090 (915050 to 1721009)	62489 (0 to 367393)	-95.08	3214.34 (2343.04 to 4303.42)	112.91 (0 to 663.87)	-6.58 (-9.82 to -3.21)
	Philippines	255183 (216526 to 294905)	11893 (3627 to 30472)	-95.34	392.77 (320.03 to 456.83)	10.67 (3.26 to 27.32)	-11.91 (-12.88 to -10.93)
	Singapore	0 (0 to 0)	0 (0 to 0)	–	0 (0 to 0)	0 (0 to 0)	–
	Thailand	844617 (755869 to 936231)	6647 (225 to 12068)	-99.21	1487.55 (1331.34 to 1648.77)	8.6 (0.29 to 15.62)	-15.61 (-16.96 to -14.23)
	Viet Nam	155271 (64415 to 318704)	17600 (5902 to 35551)	-88.66	246.17 (102.31 to 504.18)	17.23 (5.78 to 34.81)	-11.1 (-12.63 to -9.53)
**Rabies**	Global	24745 (9202 to 40729)	14076 (6124 to 21618)	-43.12	0.46 (0.18 to 0.75)	0.18 (0.08 to 0.28)	-3.35 (-3.62 to -3.08)
	China	1373 (934 to 3394)	753 (277 to 1018)	-45.18	0.13 (0.09 to 0.32)	0.05 (0.02 to 0.06)	-1.13 (-2.85 to 0.61)
	Brunei	0 (0 to 0)	0 (0 to 0)	-93.78	0.03 (0 to 0.07)	0 (0 to 0)	-15.12 (-17.84 to -12.32)
	Cambodia	118 (3 to 224)	38 (1 to 69)	-67.87	1.13 (0.03 to 1.91)	0.24 (0.01 to 0.44)	-6.7 (-7.39 to -6)
	Indonesia	481 (21 to 779)	125 (10 to 190)	-74.11	0.31 (0.01 to 0.53)	0.06 (0 to 0.09)	-6.06 (-6.32 to -5.81)
	Laos	53 (1 to 112)	21 (1 to 41)	-59.79	1.22 (0.04 to 2.31)	0.32 (0.01 to 0.6)	-4.55 (-5.14 to -3.95)
	Malaysia	0 (0 to 1)	0 (0 to 0)	-47.53	0 (0 to 0)	0 (0 to 0)	-2.84 (-3.59 to -2.09)
	Myanmar	1450 (316 to 2624)	561 (149 to 1034)	-61.33	3.2 (0.88 to 5.9)	1.04 (0.3 to 1.91)	-3.68 (-3.73 to -3.63)
	Philippines	847 (237 to 1040)	388 (175 to 596)	-54.19	1.31 (0.47 to 1.81)	0.37 (0.18 to 0.64)	-5.32 (-5.85 to -4.78)
	Singapore	0 (0 to 0)	0 (0 to 0)	-48.96	0 (0 to 0)	0 (0 to 0)	-6.79 (-8.77 to -4.78)
	Thailand	25 (2 to 46)	7 (1 to 11)	-73.45	0.06 (0 to 0.12)	0.01 (0 to 0.01)	-9.19 (-10.48 to -7.89)
	Viet Nam	215 (69 to 381)	93 (37 to 157)	-56.69	0.36 (0.14 to 0.62)	0.11 (0.04 to 0.18)	-4.44 (-4.55 to -4.34)

Supplementary Table 2. EAPCs and changes between 1990 and 2019 of Cystic echinococcosis, Dengue, Malaria and Rabies mortality in China and ASEAN countries.

		Number			Age-standardized Rate (per 100,000)		
		1990	2019	Change (%)	1990	2019	EAPC (%)
**Cystic echinococcosis**	Global	2839 (2218 to 3497)	1349 (987 to 1762)	-52.47	0.056 (0.044 to 0.069)	0.017 (0.013 to 0.022)	-4.64 (-4.86 to -4.42)
	China	72 (59 to 86)	71 (58 to 85)	-1.27	0.007 (0.006 to 0.009)	0.004 (0.003 to 0.005)	-5.52 (-6.98 to -4.02)
	Brunei	0 (0 to 0)	0 (0 to 0)	–	0 (0 to 0)	0.001 (0 to 0.001)	–
	Cambodia	0 (0 to 0)	1 (0 to 1)	–	0 (0 to 0)	0.005 (0.003 to 0.008)	–
	Indonesia	0 (0 to 0)	41 (25 to 61)	–	0 (0 to 0)	0.02 (0.012 to 0.029)	–
	Laos	2 (1 to 4)	1 (0 to 1)	-74.98	0.07 (0.038 to 0.108)	0.011 (0.006 to 0.017)	-6.32 (-6.42 to -6.21)
	Malaysia	2 (1 to 3)	1 (1 to 2)	-44.46	0.02 (0.011 to 0.03)	0.005 (0.003 to 0.007)	-5.03 (-5.22 to -4.85)
	Myanmar	24 (16 to 35)	6 (3 to 8)	-77.39	0.071 (0.047 to 0.103)	0.011 (0.007 to 0.017)	-6.56 (-6.84 to -6.29)
	Philippines	0 (0 to 0)	2 (1 to 3)	–	0 (0 to 0)	0.002 (0.001 to 0.004)	–
	Singapore	0 (0 to 0)	0 (0 to 0)	–	0 (0 to 0)	0 (0 to 0)	–
	Thailand	8 (5 to 12)	3 (2 to 5)	-62.27	0.02 (0.012 to 0.028)	0.003 (0.002 to 0.005)	-6.35 (-6.56 to -6.14)
	Viet Nam	0 (0 to 0)	6 (4 to 10)	–	0 (0 to 0)	0.007 (0.004 to 0.011)	–
**Dengue**	Global	28151 (6384 to 43024)	36055 (9176 to 44468)	28.08	0.5 (0.12 to 0.74)	0.48 (0.12 to 0.59)	0.1 (-0.09 to 0.3)
	China	68 (12 to 88)	13 (7 to 27)	-80.75	0.01 (0 to 0.01)	0 (0 to 0)	-5.8 (-6.47 to -5.13)
	Brunei	0 (0 to 1)	1 (0 to 1)	134.02	0.24 (0.13 to 0.38)	0.26 (0.12 to 0.37)	0.51 (0.34 to 0.68)
	Cambodia	70 (18 to 184)	53 (26 to 99)	-24.04	0.5 (0.16 to 1.18)	0.33 (0.17 to 0.62)	-1.93 (-3.84 to 0.01)
	Indonesia	16522 (3357 to 27087)	10074 (3101 to 13329)	-39.03	8.11 (1.81 to 12.86)	4.71 (1.49 to 6.23)	-1.83 (-1.98 to -1.69)
	Laos	22 (4 to 63)	15 (4 to 32)	-31.43	0.39 (0.09 to 1.07)	0.21 (0.06 to 0.44)	-1.42 (-2.73 to -0.09)
	Malaysia	189 (91 to 291)	274 (133 to 463)	45.44	1.16 (0.59 to 1.71)	0.92 (0.48 to 1.53)	-1.37 (-2.2 to -0.53)
	Myanmar	880 (189 to 2203)	366 (165 to 615)	-58.37	1.71 (0.42 to 3.99)	0.73 (0.33 to 1.22)	-3.11 (-3.4 to -2.81)
	Philippines	835 (315 to 1103)	2174 (901 to 2638)	160.31	1.06 (0.53 to 1.41)	1.87 (0.82 to 2.25)	2.42 (1.89 to 2.95)
	Singapore	0 (0 to 0)	5 (0 to 7)	11988.74	0 (0 to 0)	0.07 (0.01 to 0.09)	16.56 (13.9 to 19.28)
	Thailand	442 (87 to 768)	212 (97 to 324)	-52.04	0.78 (0.17 to 1.38)	0.36 (0.15 to 0.52)	-3.12 (-3.39 to -2.85)
	Viet Nam	115 (33 to 276)	122 (52 to 265)	6.05	0.16 (0.05 to 0.36)	0.14 (0.06 to 0.31)	-0.31 (-1.21 to 0.59)
**Malaria**	Global	840553 (463325 to 1356074)	643381 (301601 to 1153664)	-23.46	14.42 (7.95 to 23.21)	8.95 (4.23 to 16)	-1.65 (-2.13 to -1.17)
	China	1535 (0 to 18121)	0 (0 to 0)	-100	0.13 (0 to 1.53)	0 (0 to 0)	-44.33 (-49.43 to -38.71)
	Brunei	0 (0 to 0)	0 (0 to 0)	–	0 (0 to 0)	0 (0 to 0)	–
	Cambodia	688 (253 to 1591)	57 (10 to 159)	-91.69	6.26 (2.34 to 14.65)	0.36 (0.06 to 0.99)	-8.13 (-10.24 to -5.98)
	Indonesia	3343 (420 to 16941)	637 (161 to 1824)	-80.95	2 (0.24 to 10.26)	0.25 (0.06 to 0.72)	-4.52 (-6.71 to -2.29)
	Laos	108 (40 to 244)	23 (5 to 61)	-78.97	2.74 (1 to 6.22)	0.34 (0.08 to 0.93)	-7.5 (-9.94 to -5)
	Malaysia	132 (46 to 345)	5 (1 to 17)	-96.01	0.82 (0.28 to 2.12)	0.02 (0 to 0.05)	-14.64 (-16.06 to -13.19)
	Myanmar	5070 (1601 to 13582)	294 (0 to 1682)	-94.2	12.11 (3.79 to 31.84)	0.55 (0 to 3.13)	-4.82 (-8.98 to -0.48)
	Philippines	905 (177 to 2162)	46 (7 to 157)	-94.96	1.56 (0.34 to 3.77)	0.04 (0.01 to 0.14)	-12.5 (-14.88 to -10.05)
	Singapore	0 (0 to 0)	0 (0 to 0)	–	0 (0 to 0)	0 (0 to 0)	–
	Thailand	1375 (582 to 2785)	1 (0 to 3)	-99.92	2.46 (1.04 to 4.98)	0 (0 to 0)	-18.05 (-21.01 to -14.98)
	Viet Nam	694 (168 to 2271)	59 (10 to 140)	-91.43	1.19 (0.29 to 3.93)	0.06 (0.01 to 0.14)	-10.66 (-12.77 to -8.5)
**Rabies**	Global	26029 (9747 to 40716)	13743 (6019 to 17939)	-47.2	0.48 (0.18 to 0.74)	0.18 (0.08 to 0.24)	-3.63 (-3.9 to -3.35)
	China	1305 (972 to 2756)	719 (264 to 898)	-44.91	0.12 (0.09 to 0.26)	0.04 (0.02 to 0.05)	-0.88 (-2.84 to 1.11)
	Brunei	0 (0 to 0)	0 (0 to 0)	-93.36	0.03 (0 to 0.07)	0 (0 to 0)	-14.97 (-17.9 to -11.93)
	Cambodia	138 (3 to 290)	41 (1 to 74)	-70.55	1.27 (0.03 to 2.44)	0.27 (0.01 to 0.47)	-6.81 (-7.56 to -6.06)
	Indonesia	510 (24 to 758)	134 (11 to 195)	-73.68	0.31 (0.01 to 0.49)	0.07 (0 to 0.1)	-5.87 (-6.17 to -5.57)
	Laos	59 (1 to 124)	20 (1 to 37)	-65.79	1.32 (0.04 to 2.54)	0.31 (0.01 to 0.56)	-4.89 (-5.45 to -4.32)
	Malaysia	0 (0 to 1)	0 (0 to 0)	-49.03	0 (0 to 0)	0 (0 to 0)	-3.06 (-3.7 to -2.41)
	Myanmar	1575 (370 to 2778)	504 (148 to 953)	-67.99	3.43 (1.04 to 5.94)	0.93 (0.31 to 1.72)	-4.45 (-4.57 to -4.32)
	Philippines	904 (262 to 1072)	378 (185 to 526)	-58.18	1.36 (0.5 to 1.57)	0.36 (0.2 to 0.56)	-5.6 (-6.18 to -5.02)
	Singapore	0 (0 to 0)	0 (0 to 0)	-51.9	0 (0 to 0)	0 (0 to 0)	-6.97 (-10.09 to -3.74)
	Thailand	25 (2 to 43)	7 (2 to 10)	-72.94	0.06 (0 to 0.11)	0.01 (0 to 0.01)	-9.38 (-10.8 to -7.94)
	Viet Nam	210 (76 to 376)	107 (49 to 170)	-49	0.36 (0.15 to 0.6)	0.12 (0.05 to 0.2)	-4 (-4.12 to -3.88)

Supplementary Table 3. Join-point regression results, periods with uptrends of ASIRs and ASMR of Cystic echinococcosis, Dengue, Malaria

and Rabies.

location	Cause	Start Year	End Year	APC (95% CI)	P Value
ASIR					
Global	Cystic echinococcosis	1990	1993	0.7 (0.6 to 0.8)	<0.001
	Dengue	1990	1998	1.3 (1.2 to 1.4)	<0.001
	Malaria	1994	2007	0 (0 to 0.1)	0.224
	Dengue	1998	2007	2 (2 to 2.1)	<0.001
	Cystic echinococcosis	2005	2019	0.2 (0.2 to 0.2)	<0.001
	Dengue	2007	2010	1.6 (1.1 to 2)	<0.001
	Malaria	2015	2019	0.6 (0 to 1.2)	0.035
China	Dengue	1990	1995	2.6 (2.4 to 2.8)	<0.001
	Dengue	1995	1999	4.7 (4.3 to 5.2)	<0.001
	Cystic echinococcosis	1998	2001	0.4 (0.3 to 0.5)	<0.001
	Dengue	1999	2009	6.5 (6.3 to 6.7)	<0.001
	Rabies	2000	2005	18.3 (16.3 to 20.3)	<0.001
	Cystic echinococcosis	2001	2004	2.5 (2.4 to 2.6)	<0.001
	Cystic echinococcosis	2004	2007	0.5 (0.4 to 0.6)	<0.001
	Cystic echinococcosis	2007	2019	0.3 (0.3 to 0.3)	<0.001
	Dengue	2014	2017	10.4 (7.2 to 13.6)	<0.001
Brunei	Rabies	1990	1996	4.4 (1.7 to 7.2)	0.004
	Dengue	2000	2005	4 (3.5 to 4.6)	<0.001
	Dengue	2005	2010	0.8 (0.5 to 1.2)	<0.001
	Rabies	2010	2014	18.8 (12.4 to 25.5)	<0.001
Cambodia	Cystic echinococcosis	1990	1994	1.6 (1.6 to 1.7)	<0.001
	Rabies	1990	1996	0.2 (0 to 0.5)	0.081
	Malaria	1994	2001	4.2 (1.8 to 6.8)	0.002
	Dengue	2000	2005	6.7 (6.2 to 7.1)	<0.001

	Dengue	2017	2019	5.1 (3.7 to 6.5)	<0.001
Indonesia	Cystic echinococcosis	1990	1998	0.3 (0.2 to 0.3)	<0.001
	Dengue	1999	2005	2.2 (1.8 to 2.6)	<0.001
	Cystic echinococcosis	2005	2019	0.1 (0.1 to 0.1)	<0.001
	Malaria	2005	2008	29.1 (20.6 to 38.2)	<0.001
	Malaria	2008	2011	7.1 (0.5 to 14.1)	0.036
	Dengue	2010	2014	1 (0.7 to 1.4)	<0.001
	Dengue	2017	2019	0.9 (0.3 to 1.6)	0.005
Laos	Cystic echinococcosis	1990	1994	0.4 (0.4 to 0.4)	<0.001
	Dengue	1990	1994	12.5 (9.9 to 15.2)	<0.001
	Malaria	1990	1999	8.8 (7 to 10.6)	<0.001
	Dengue	2005	2010	10.2 (7.8 to 12.6)	<0.001
	Malaria	2011	2015	10.5 (1.5 to 20.3)	0.025
Malaysia	Dengue	1990	1996	0.6 (0 to 1.2)	0.036
	Rabies	1995	2003	1.8 (1.4 to 2.1)	<0.001
	Dengue	1996	2001	1.9 (1.1 to 2.7)	<0.001
	Dengue	2001	2004	3.5 (1.8 to 5.2)	0.001
	Dengue	2004	2011	1.1 (0.6 to 1.5)	<0.001
	Dengue	2011	2014	6.9 (3.6 to 10.3)	0.001
	Malaria	2015	2019	28 (11.2 to 47.4)	0.002
Myanmar	Cystic echinococcosis	1990	1994	0.4 (0.4 to 0.4)	<0.001
	Dengue	1998	2005	3.8 (3.3 to 4.4)	<0.001
	Malaria	2005	2011	9.6 (3.8 to 15.7)	0.003
	Dengue	2010	2015	2.6 (2 to 3.2)	<0.001
Philippines	Dengue	1990	2000	1.4 (1.3 to 1.6)	<0.001
	Dengue	2000	2006	6.2 (5.9 to 6.4)	<0.001
	Dengue	2006	2009	8.2 (7.8 to 8.6)	<0.001
	Dengue	2009	2016	0.7 (0.5 to 0.8)	<0.001

	Dengue	2016	2019	5.2 (4.6 to 5.8)	<0.001
Singapore	Rabies	1990	1999	2 (1.4 to 2.7)	<0.001
	Cystic echinococcosis	1996	1999	0.1 (0.1 to 0.2)	<0.001
	Dengue	2000	2005	6.9 (4 to 9.8)	<0.001
	Rabies	2005	2014	11 (9.8 to 12.1)	<0.001
	Dengue	2010	2014	7.5 (4.1 to 11)	<0.001
Thailand	Dengue	2000	2006	0.8 (0.5 to 1)	<0.001
	Dengue	2006	2010	1.9 (1.4 to 2.3)	<0.001
	Rabies	2014	2019	0.9 (0.3 to 1.5)	0.007
	Dengue	2015	2019	2.4 (2.1 to 2.7)	<0.001
Viet Nam	Dengue	1990	1994	1.3 (0.3 to 2.4)	0.014
	Malaria	1990	2001	4.7 (2.3 to 7.2)	0.001
	Dengue	2000	2005	1.7 (0.6 to 2.8)	0.005
	Cystic echinococcosis	2001	2004	1.6 (1.5 to 1.6)	<0.001
	Dengue	2014	2017	4.4 (0.3 to 8.6)	0.037
ASMR					
Global	Dengue	2005	2013	1.7 (1 to 2.5)	<0.001
	Cystic echinococcosis	2017	2019	8.6 (5.7 to 11.5)	<0.001
China	Rabies	1998	2005	20.2 (19.1 to 21.3)	<0.001
	Cystic echinococcosis	2016	2019	66.2 (35.8 to 103.5)	<0.001
Brunei	Rabies	1990	1998	3.2 (0.2 to 6.2)	0.036
	Dengue	1997	2005	1.6 (1.1 to 2)	<0.001
	Rabies	2009	2014	16.4 (10.7 to 22.4)	<0.001
	Dengue	2011	2016	2.2 (1.3 to 3.2)	<0.001
Cambodia	Dengue	1995	1998	120.7 (112 to 129.8)	<0.001
	Dengue	2001	2007	35.4 (34.3 to 36.5)	<0.001
	Dengue	2010	2019	1.2 (0.9 to 1.4)	<0.001
Indonesia	Malaria	2002	2010	18.9 (6.2 to 33.1)	0.004

Laos	Dengue	2010	2013	72.1 (62.3 to 82.4)	<0.001
	Dengue	2016	2019	13.1 (10.1 to 16.2)	<0.001
Myanmar	Malaria	1996	2009	12.3 (8.4 to 16.2)	<0.001
Philippines	Dengue	1990	2019	2.5 (2 to 3.1)	<0.001
	Malaria	2001	2007	20.2 (0.7 to 43.4)	0.042
Singapore	Dengue	1990	1997	9.4 (1.3 to 18.2)	0.025
	Rabies	1990	1999	8 (3.3 to 12.9)	0.002
	Dengue	1997	2004	45.2 (31.3 to 60.6)	<0.001
	Dengue	2004	2019	4.2 (1.4 to 7)	0.004
	Rabies	2006	2013	27.9 (14.5 to 42.9)	<0.001
Viet Nam	Malaria	1990	2000	4.9 (0.3 to 9.8)	0.039
	Dengue	1995	1998	40.7 (11.6 to 77.2)	0.006
	Dengue	2001	2019	1.1 (0.2 to 1.9)	0.014

Supplementary Figure 1. Age-standardized incidence and mortality rates of cystic echinococcosis, dengue, malaria, and rabies in China and ASEAN countries, from 1990 to 2019.

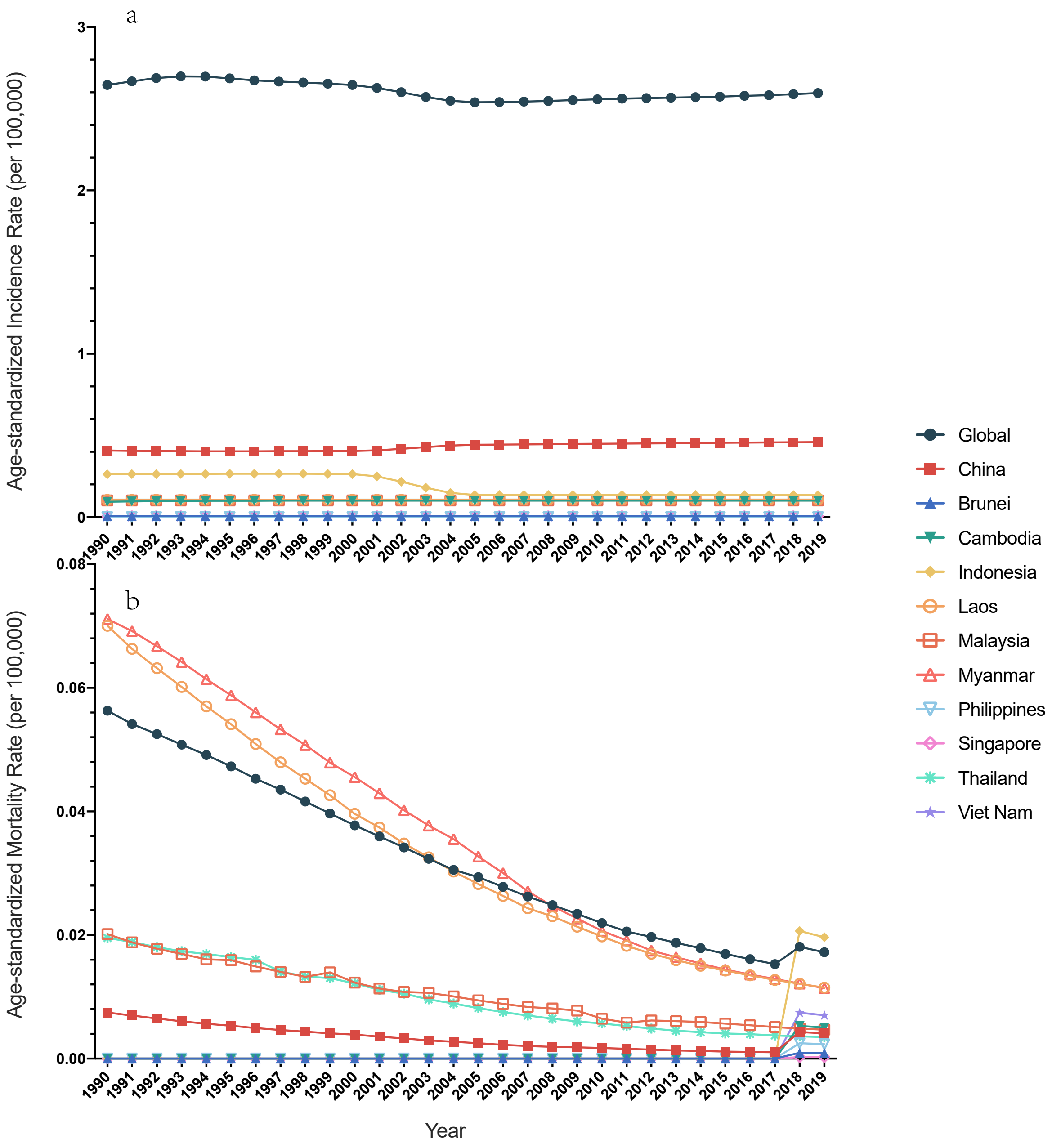

Supplementary Figure 1A. Cystic echinococcosis. a: Age-standardized Incidence rate (per 100,000) from 1990 to 2019; b: Age-standardized Mortality rate (per 100,000) from 1990 to 2019.

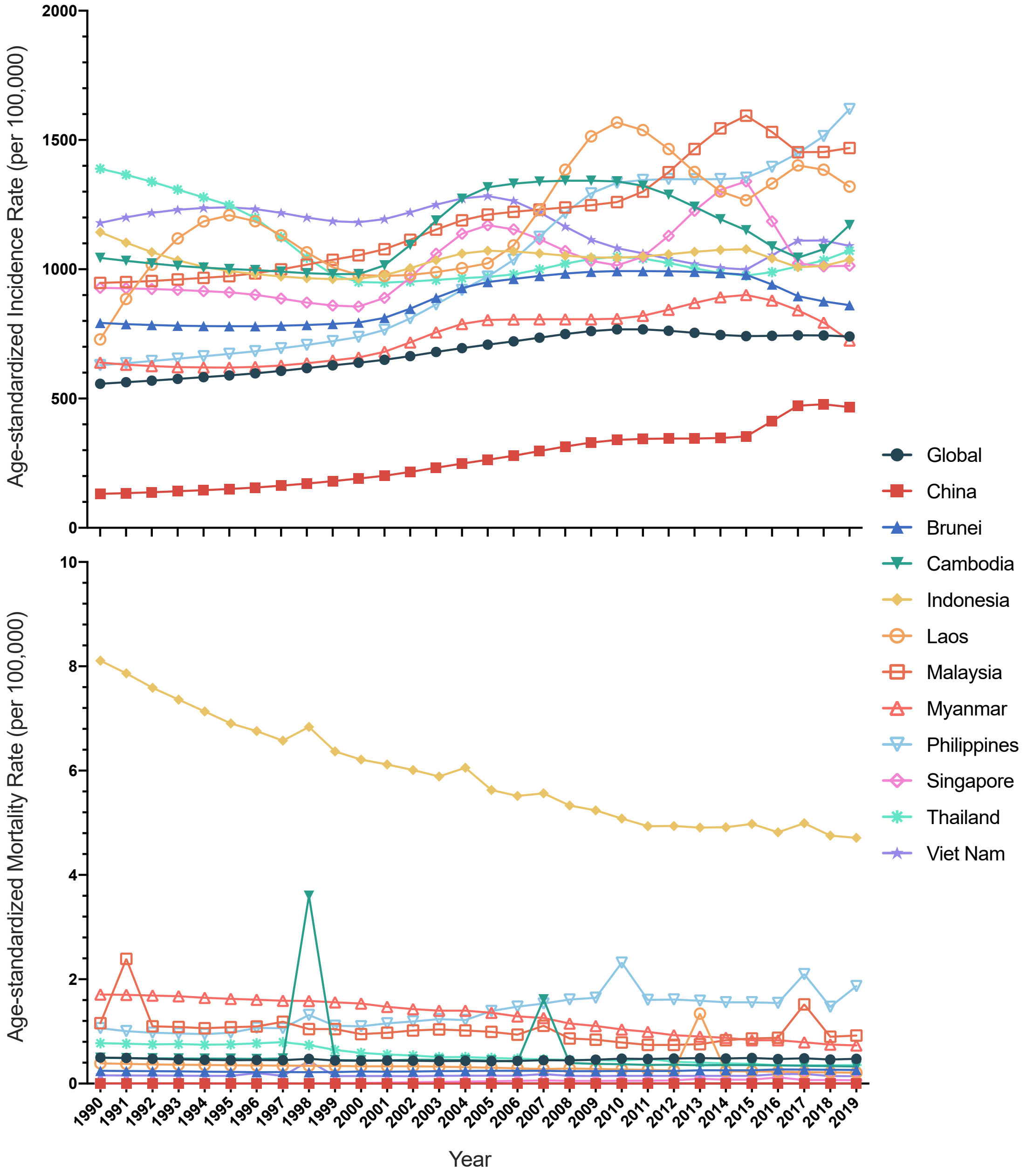

Supplementary Figure 1B. Dengue. a: Age-standardized Incidence rate (per 100,000) from 1990 to 2019; b: Age-standardized Mortality rate (per 100,000) from 1990 to 2019.

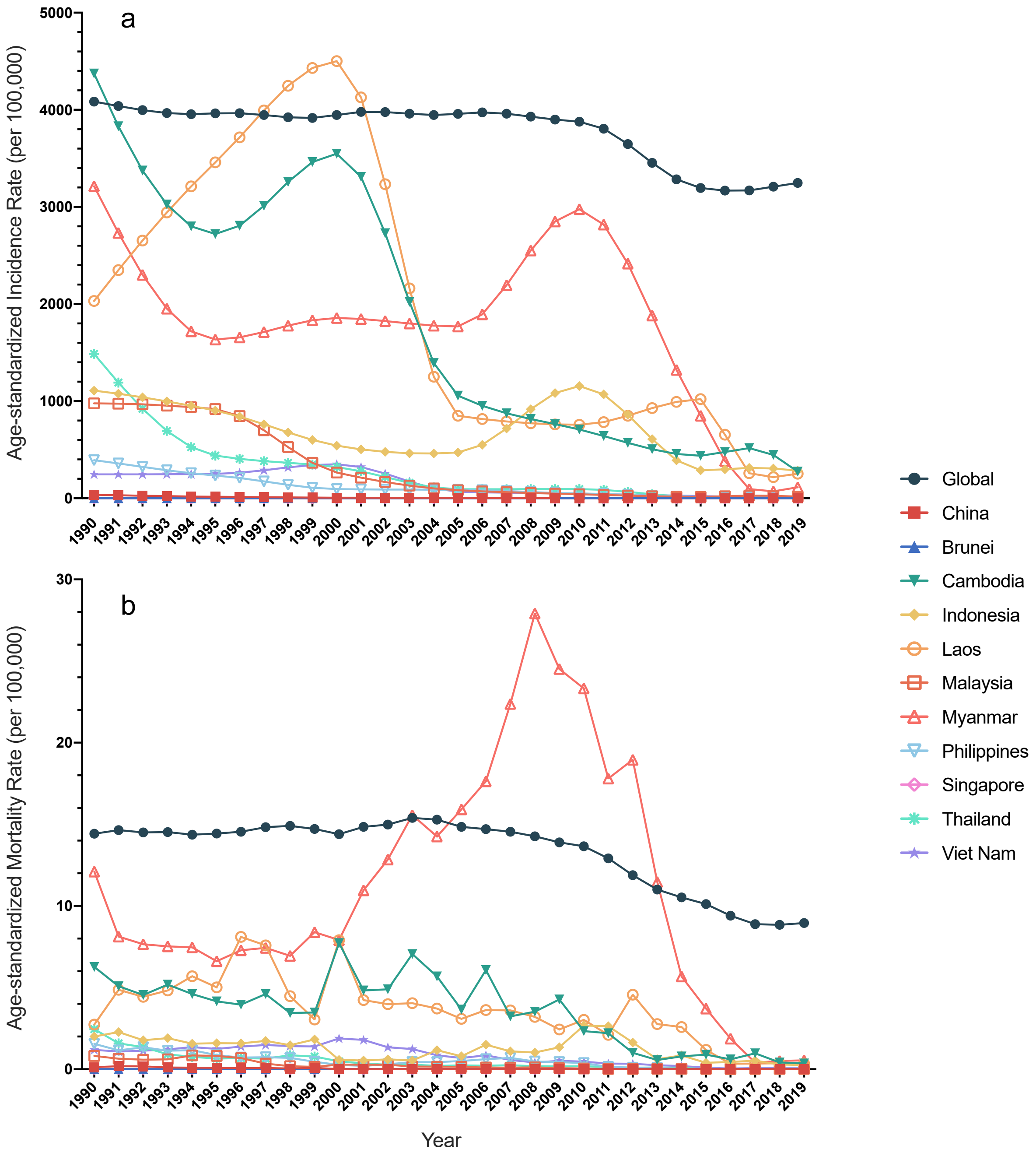

Supplementary Figure 1C. Malaria. a: Age-standardized Incidence rate (per 100,000) from 1990 to 2019; b: Age-standardized Mortality rate (per 100,000) from 1990 to 2019.

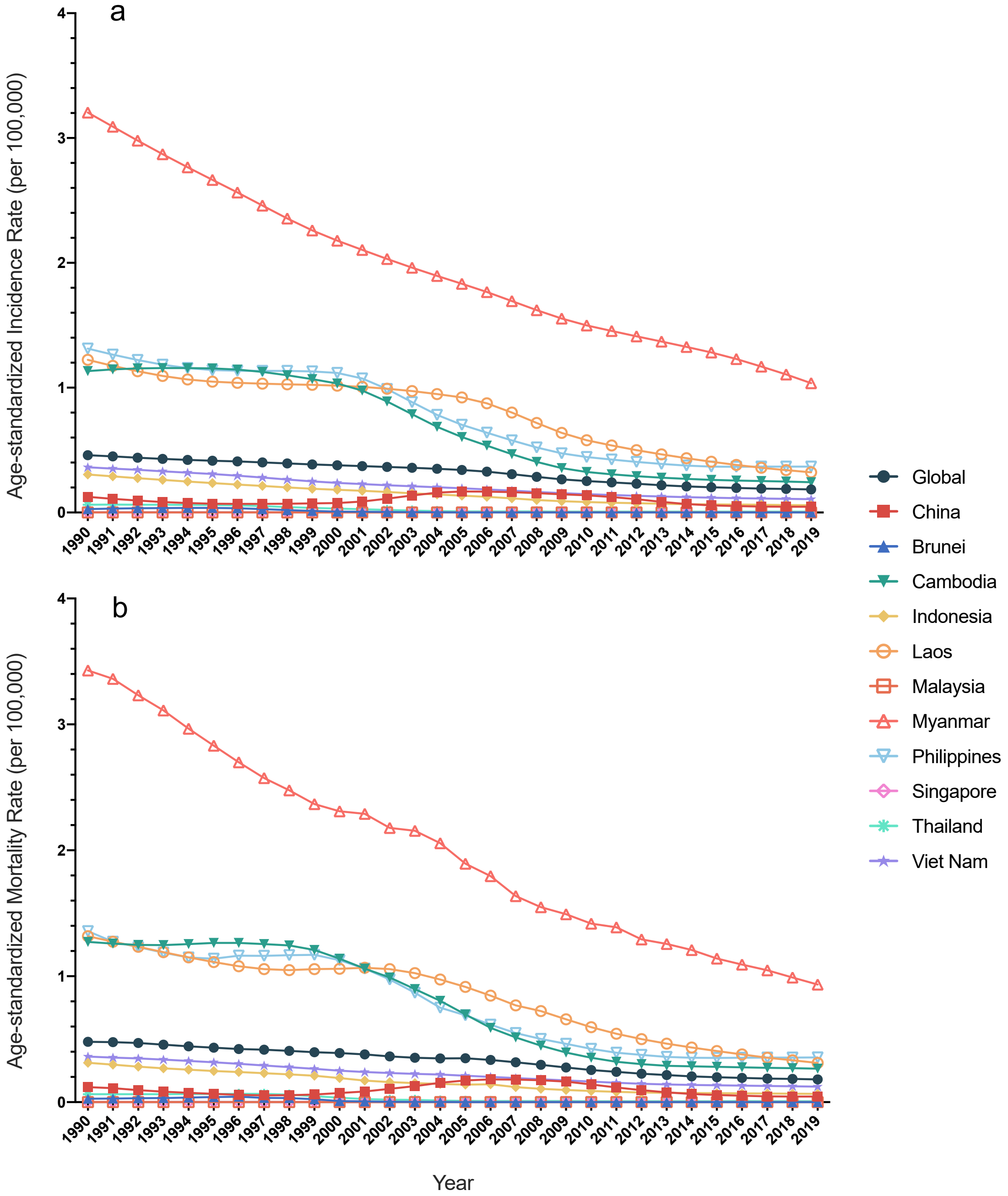

Supplementary Figure 1D. Rabies. a: Age-standardized Incidence rate (per 100,000) from 1990 to 2019; b: Age-standardized Mortality rate (per 100,000) from 1990 to 2019.

Supplementary Figure 2. Incidence and death number of cystic echinococcosis, dengue, malaria, and rabies in China and ASEAN countries, by sex, from 1990 to 2019.

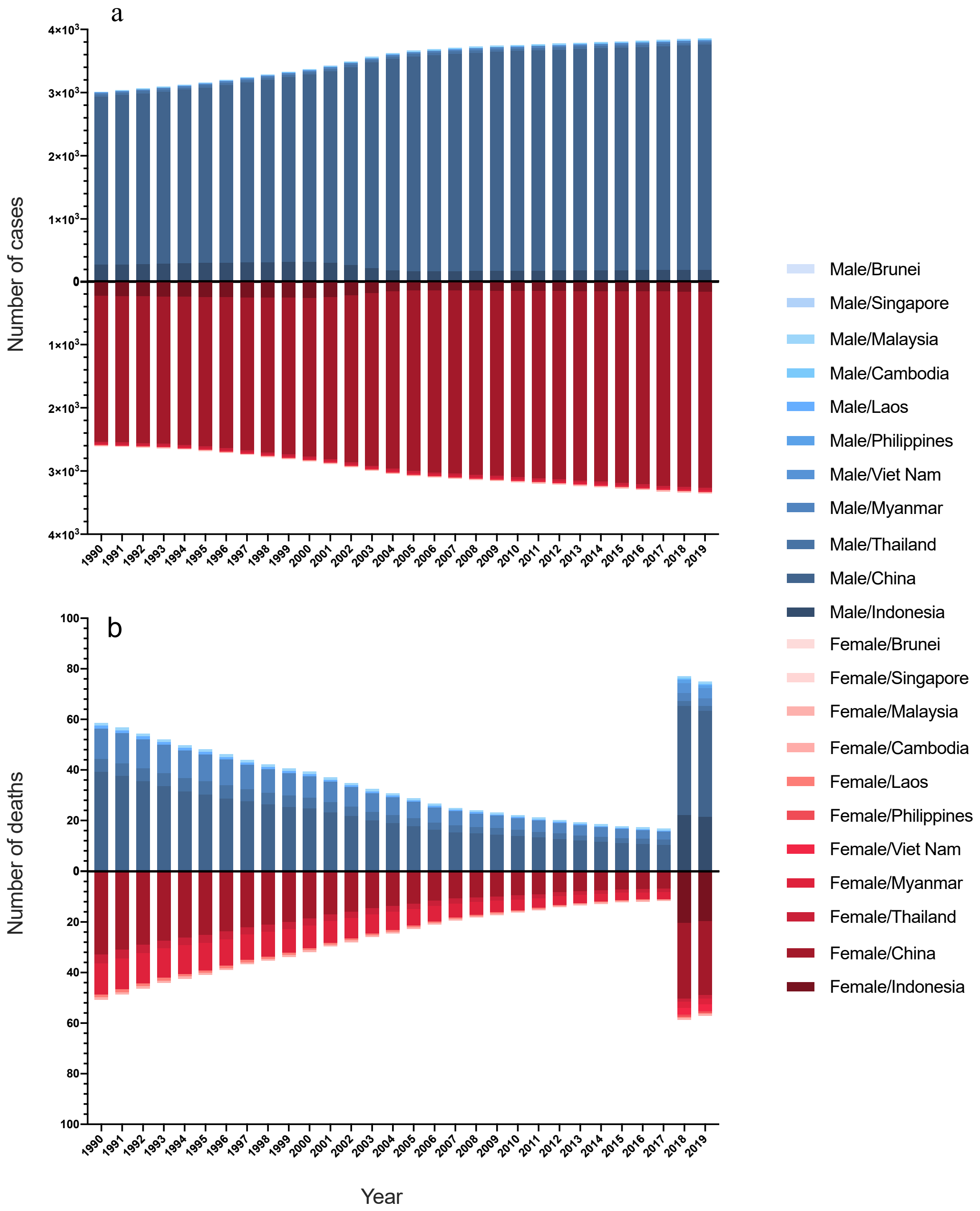

Supplementary Figure 2A. Cystic echinococcosis. a: number of cases from 1990 to 2019; b: number of deaths from 1990 to 2019.

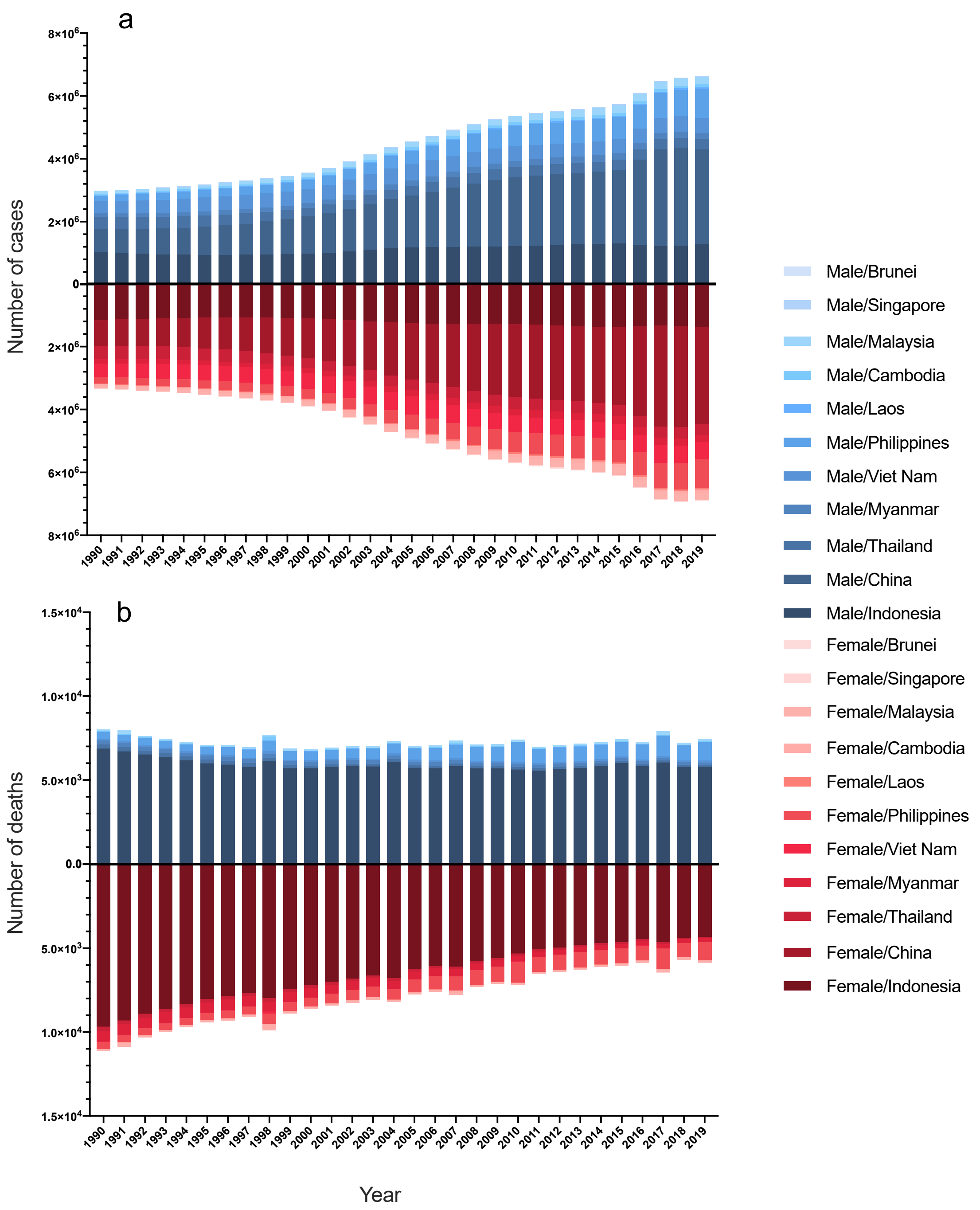

Supplementary Figure 2B. Dengue. a: number of cases from 1990 to 2019; b: number of deaths from 1990 to 2019.

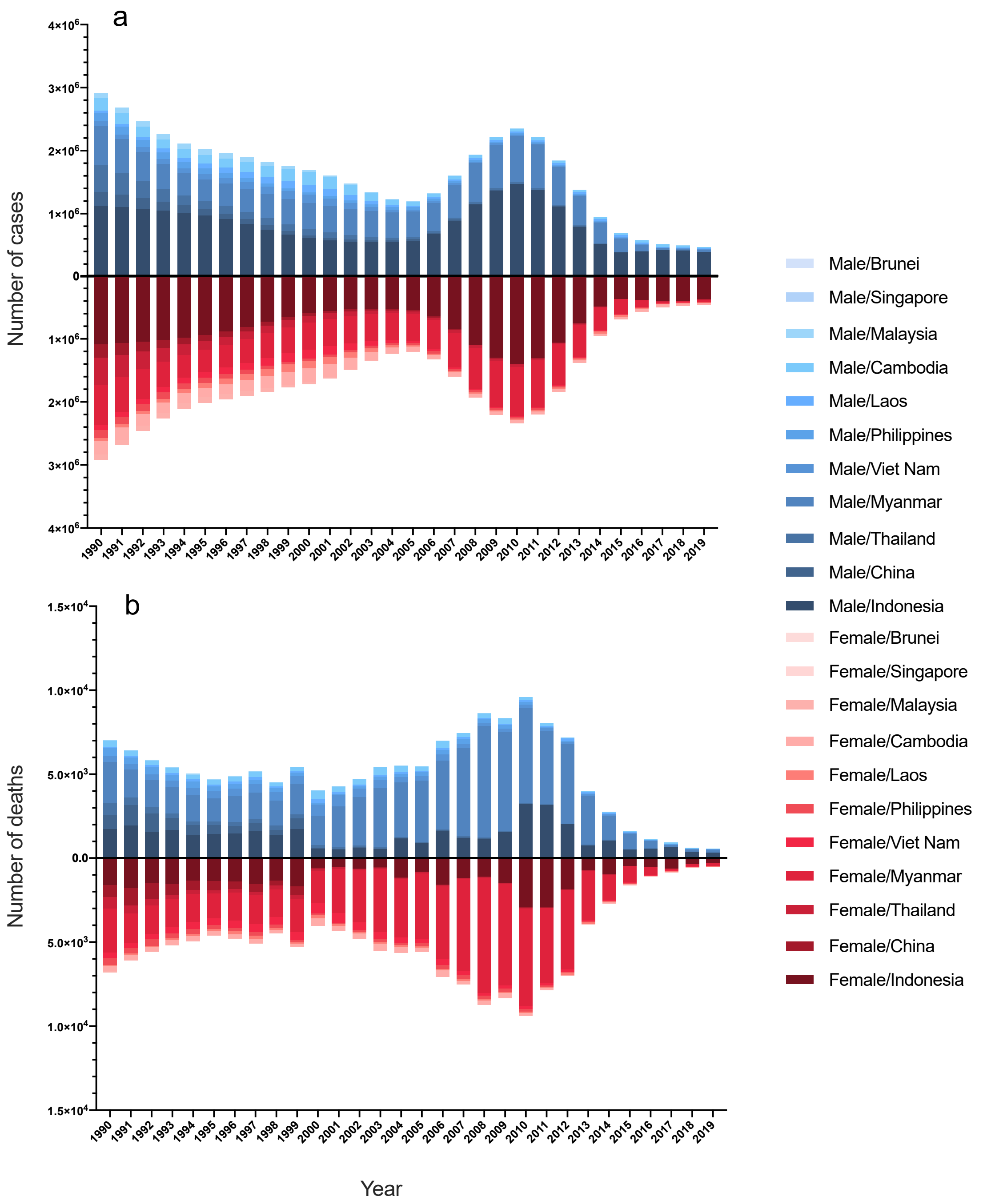

Supplementary Figure 2C. Malaria. a: number of cases from 1990 to 2019; b: number of deaths from 1990 to 2019.

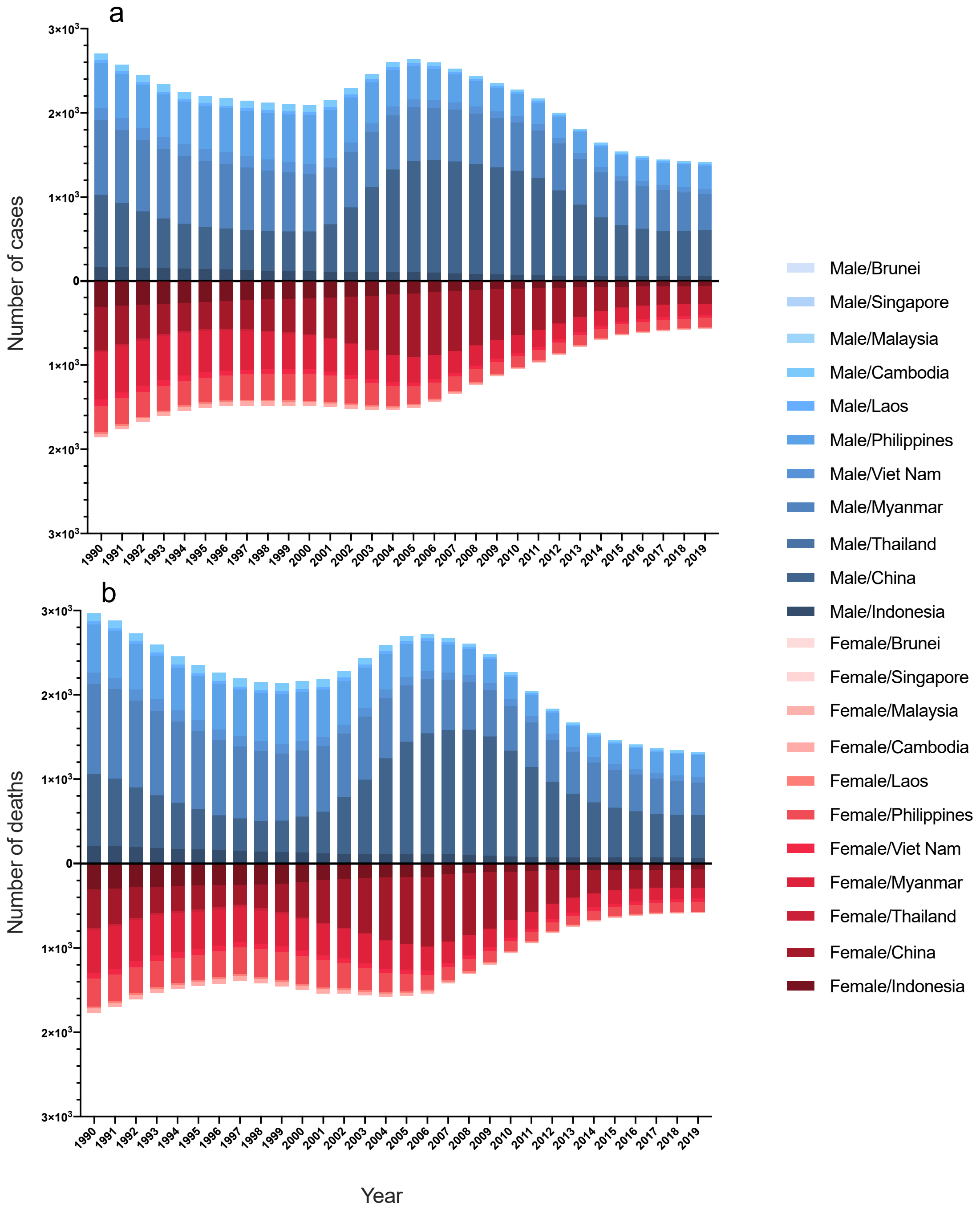

Supplementary Figure 2D. Rabies. a: number of cases from 1990 to 2019; b: number of deaths from 1990 to 2019.

Supplementary Figure 3. Age-specific incidence and mortality rates of cystic echinococcosis, dengue, malaria, and rabies in China and ASEAN countries, in 1990 and 2019.

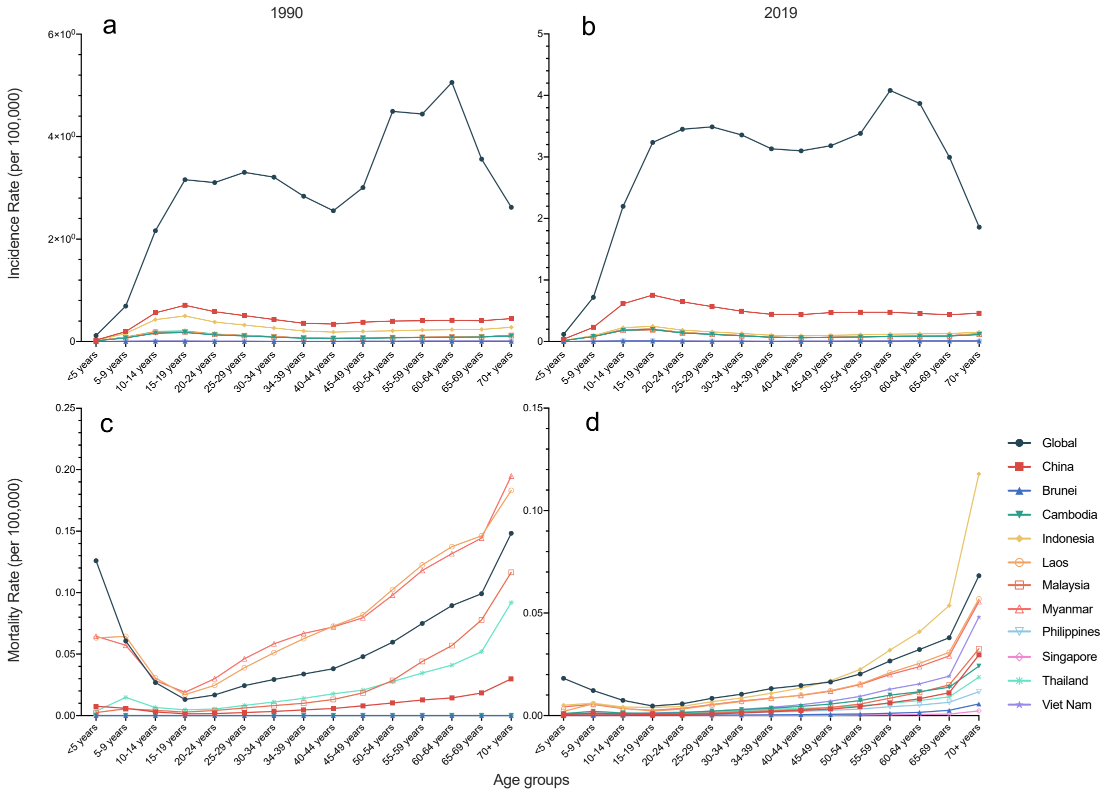

Supplementary Figure 3A. Cystic echinococcosis. a: Incidence rate (per 100,000) in 1990; b: Incidence rate (per 1990) in 2019; c: Mortality rate (per 100,000) in 1990; d: Mortality rate (per 100,000) in 2019.

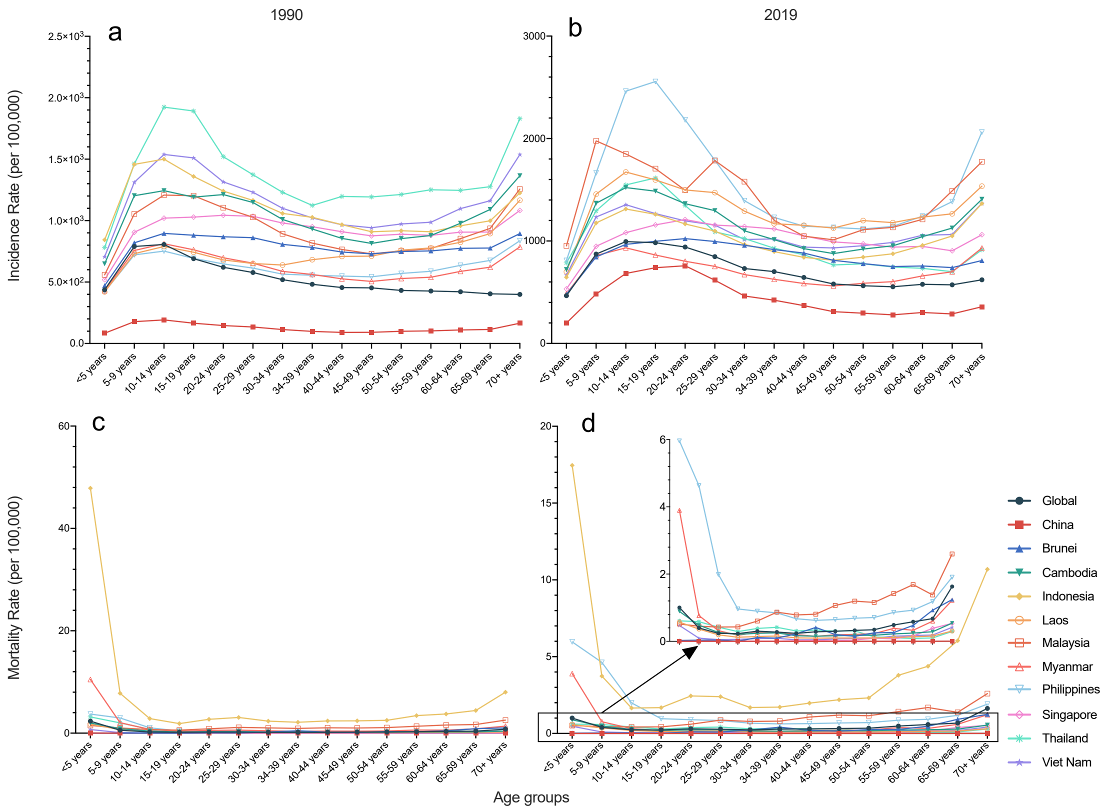

Supplementary Figure 3B. Dengue. a: Incidence rate (per 100,000) in 1990; b: Incidence rate (per 1990) in 2019; c: Mortality rate (per 100,000) in 1990; d: Mortality rate (per 100,000) in 2019.

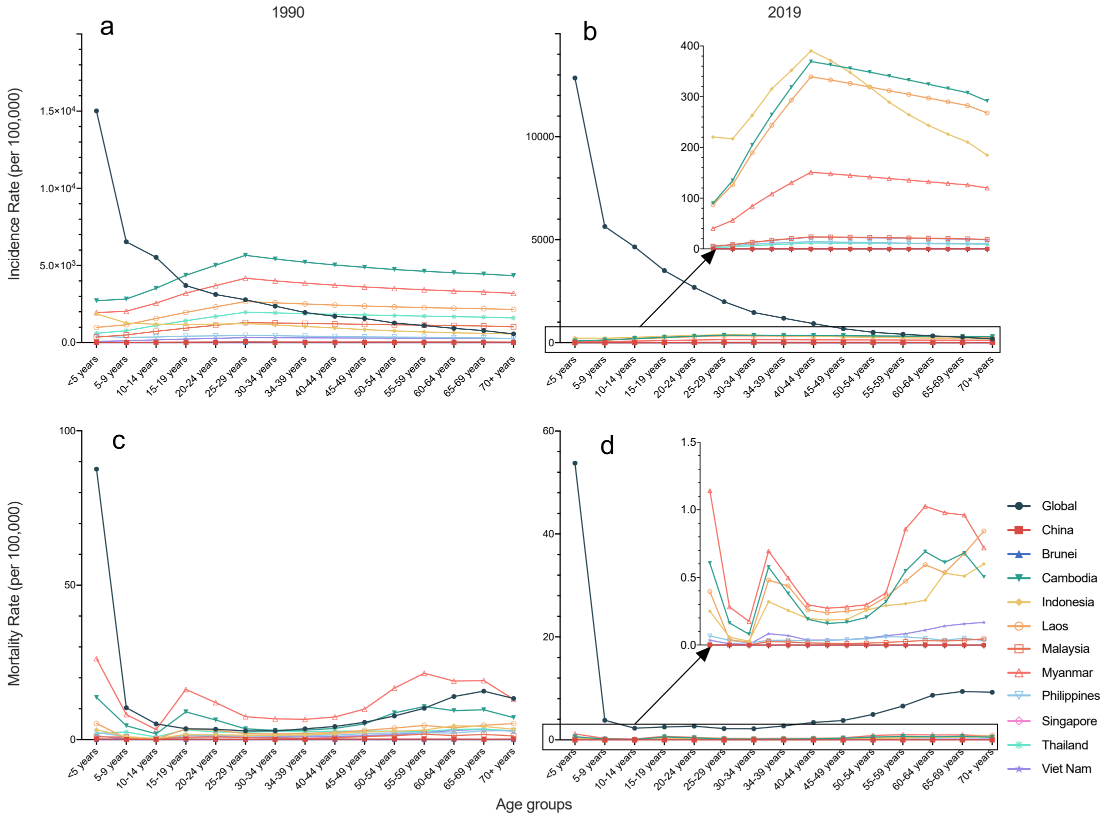

Supplementary Figure 3C. Malaria. a: Incidence rate (per 100,000) in 1990; b: Incidence rate (per 1990) in 2019; c: Mortality rate (per 100,000) in 1990; d: Mortality rate (per 100,000) in 2019.

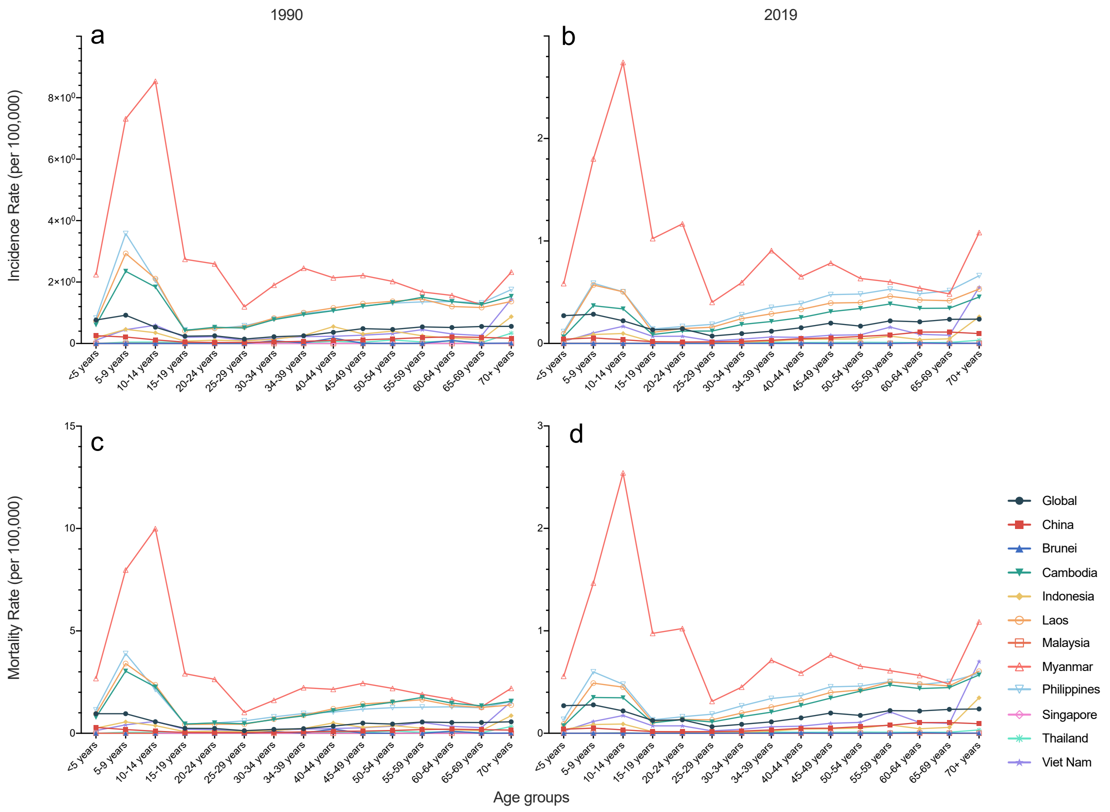

Supplementary Figure 3D. Rabies. a: Incidence rate (per 100,000) in 1990; b: Incidence rate (per 1990) in 2019; c: Mortality rate (per 100,000) in 1990; d: Mortality rate (per 100,000) in 2019.

Supplementary Figure 4. EAPC in different age groups of cystic echinococcosis, dengue, malaria, and rabies in China and ASEAN countries, and the corresponding 95% Confident Intervals, from 1990 to 2019.

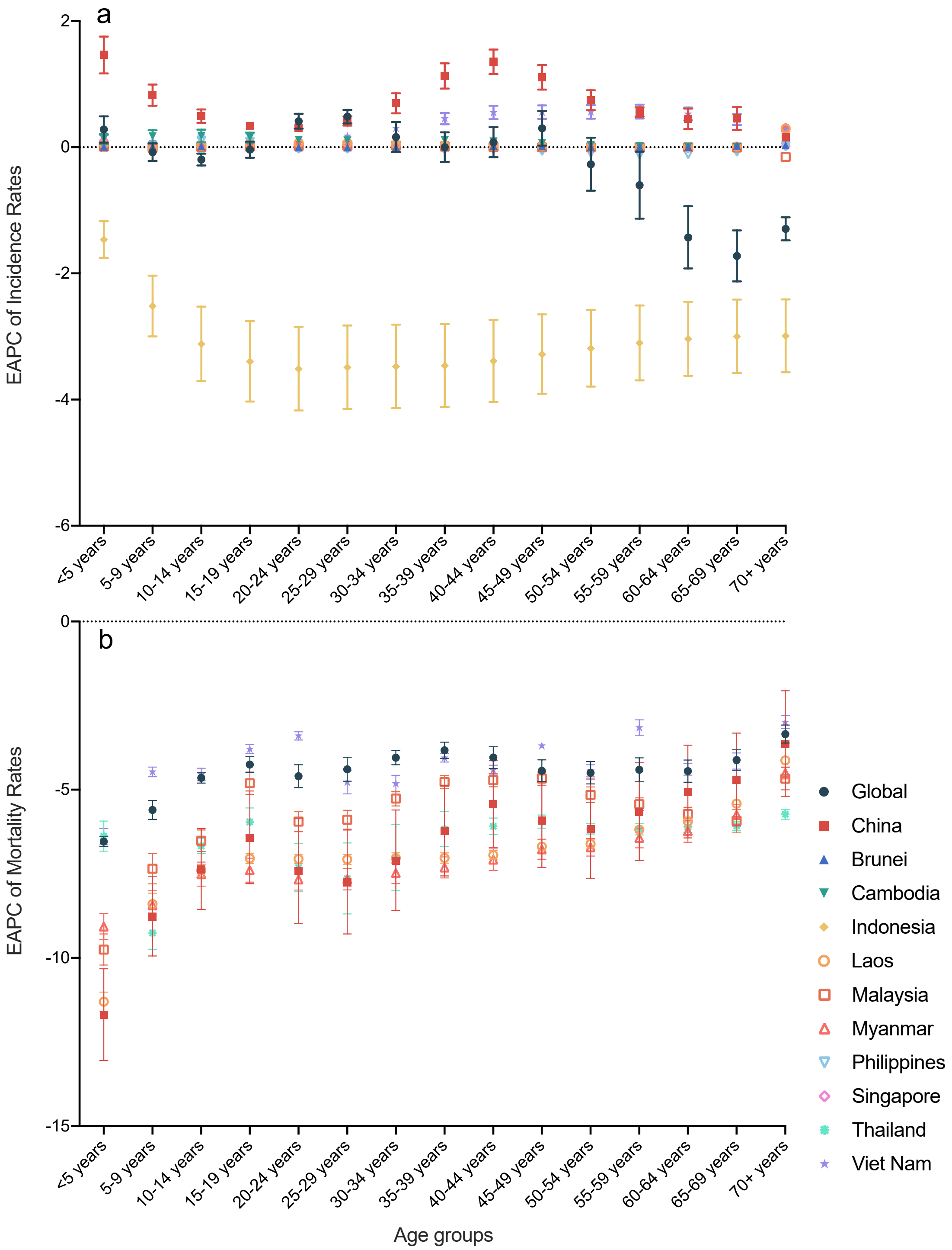

Supplementary Figure 4A. Cystic echinococcosis. a: EAPC (%) of Incidence rates; b: EAPC (%) of Mortality Rates.

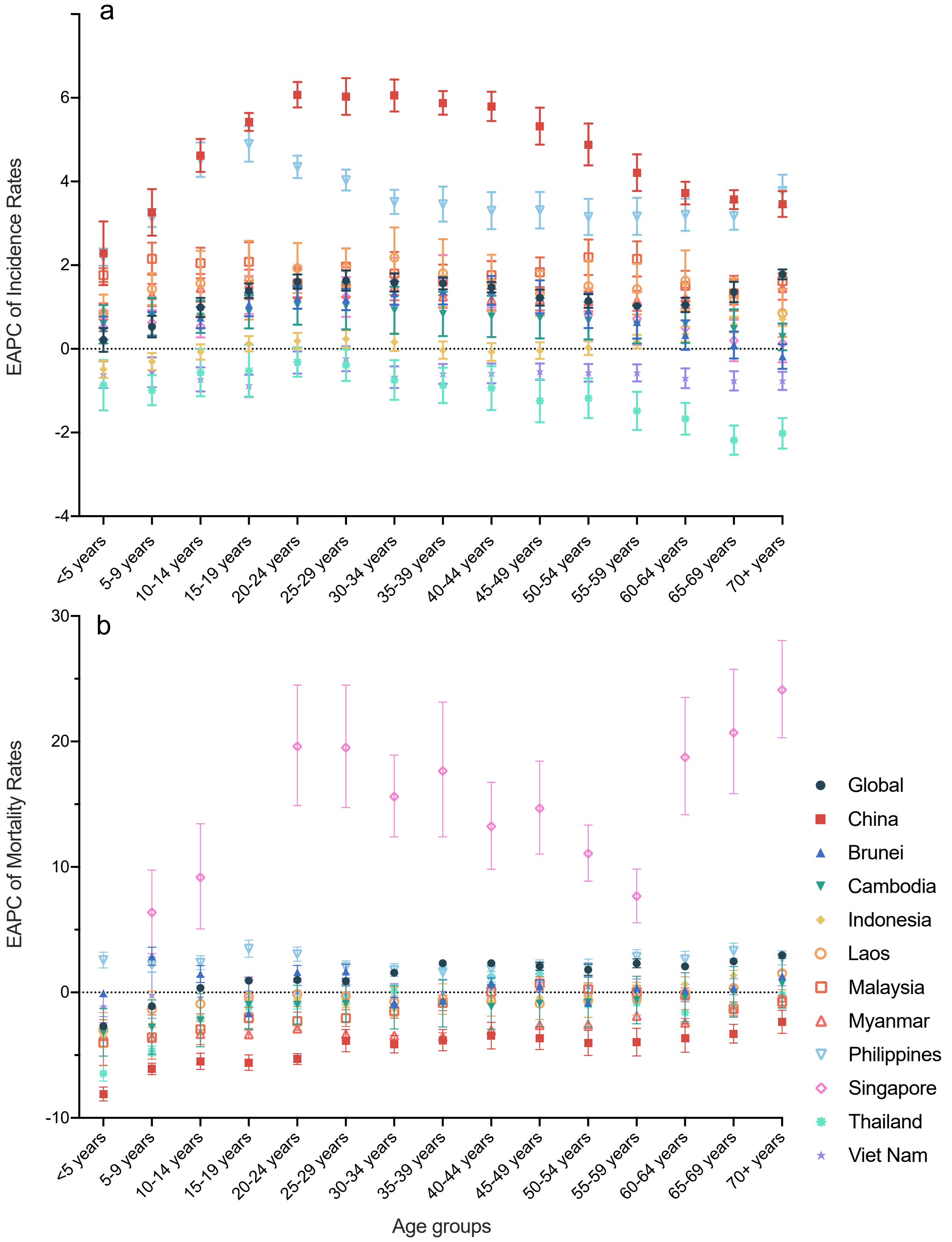

Supplementary Figure 4B. Dengue. a: EAPC (%) of Incidence rates; b: EAPC (%) of Mortality Rates.

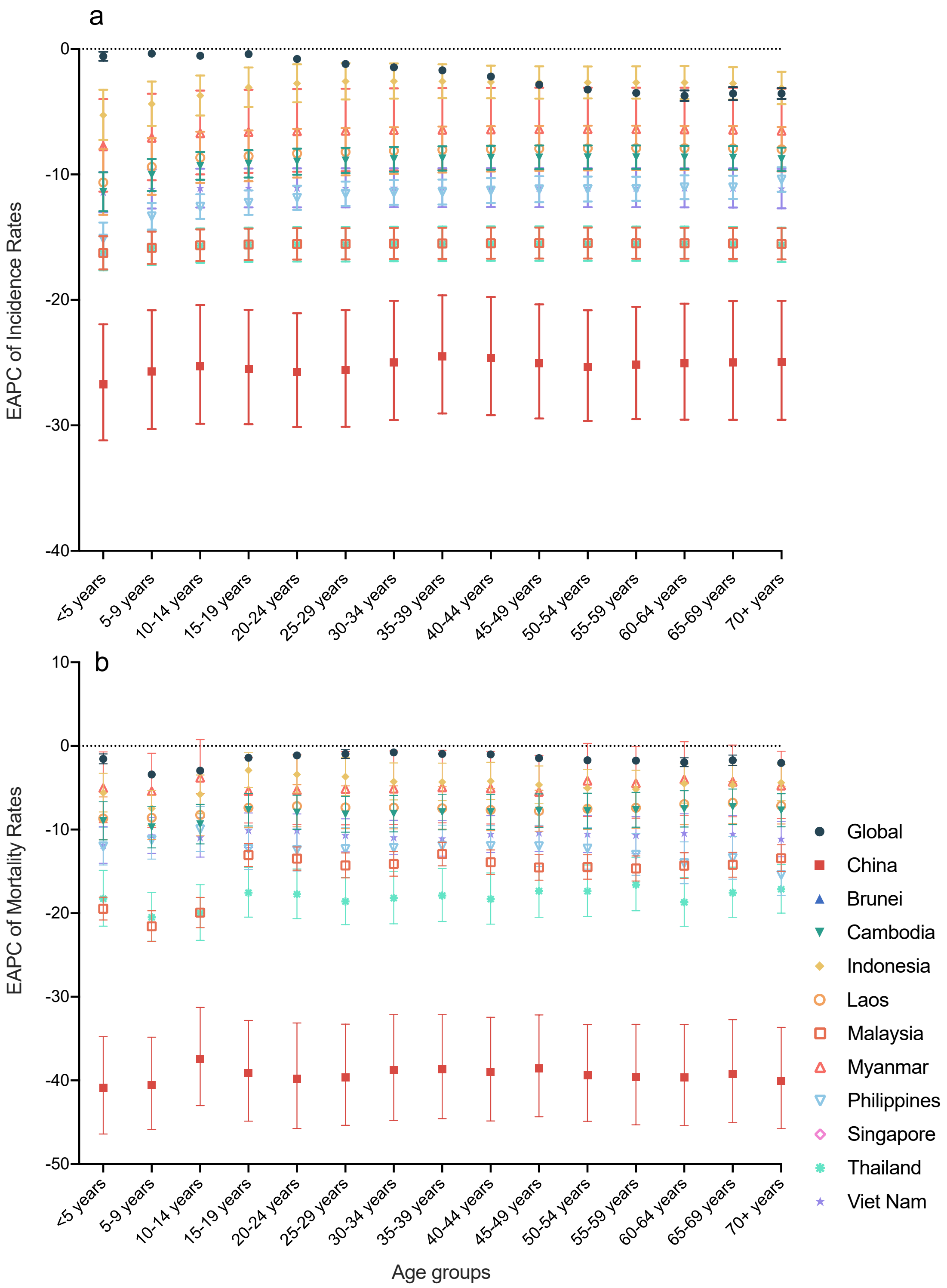

Supplementary Figure 4C. Malaria. a: EAPC (%) of Incidence rates; b: EAPC (%) of Mortality Rates.

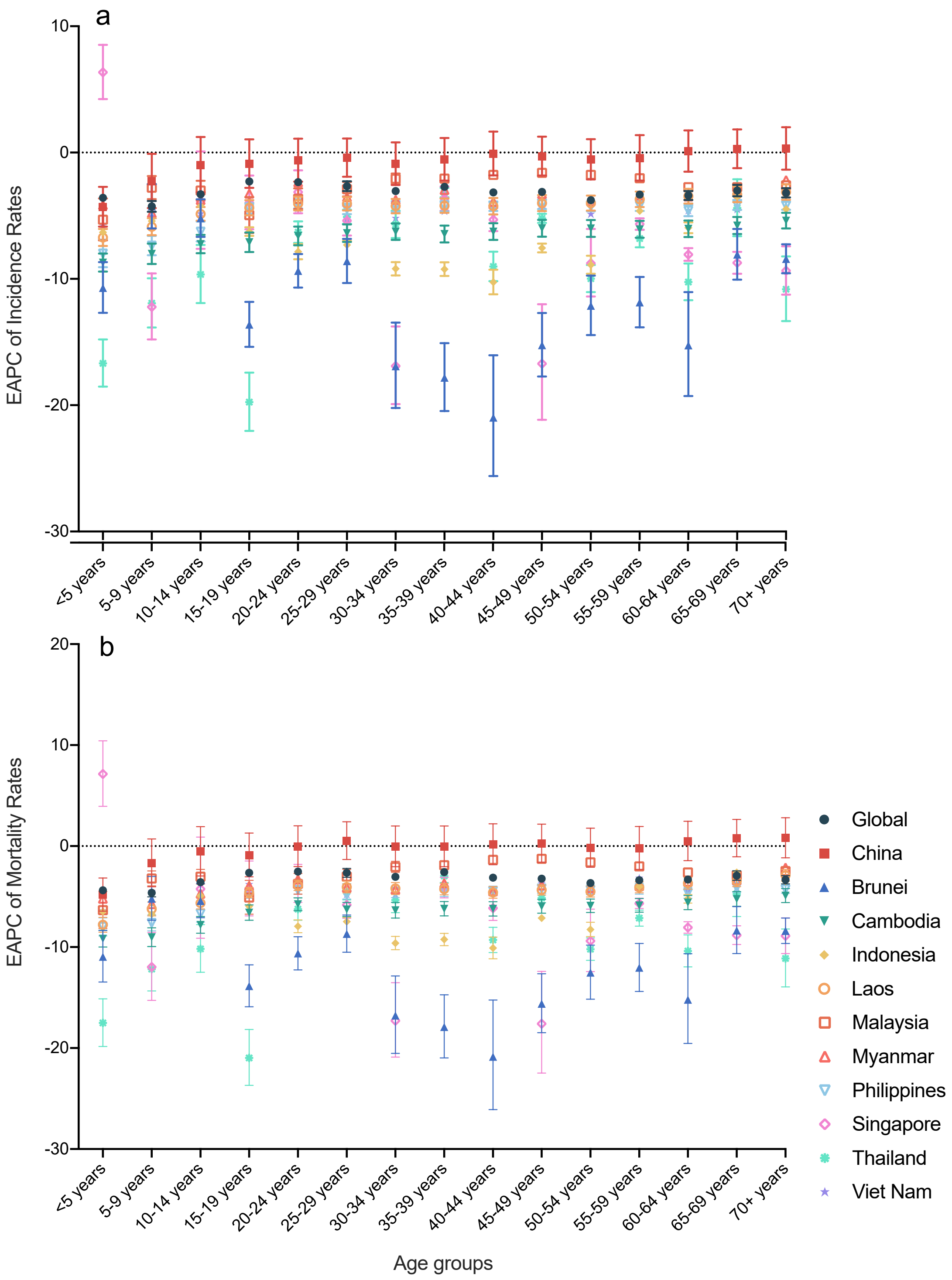

Supplementary Figure 4D. Rabies. a: EAPC (%) of Incidence rates; b: EAPC (%) of Mortality Rates.

Supplementary Figure 5. The correlation between age-standardized incidence and mortality rates of cystic echinococcosis, dengue, malaria, and rabies and Socio-demographic Index in China and ASEAN countries.

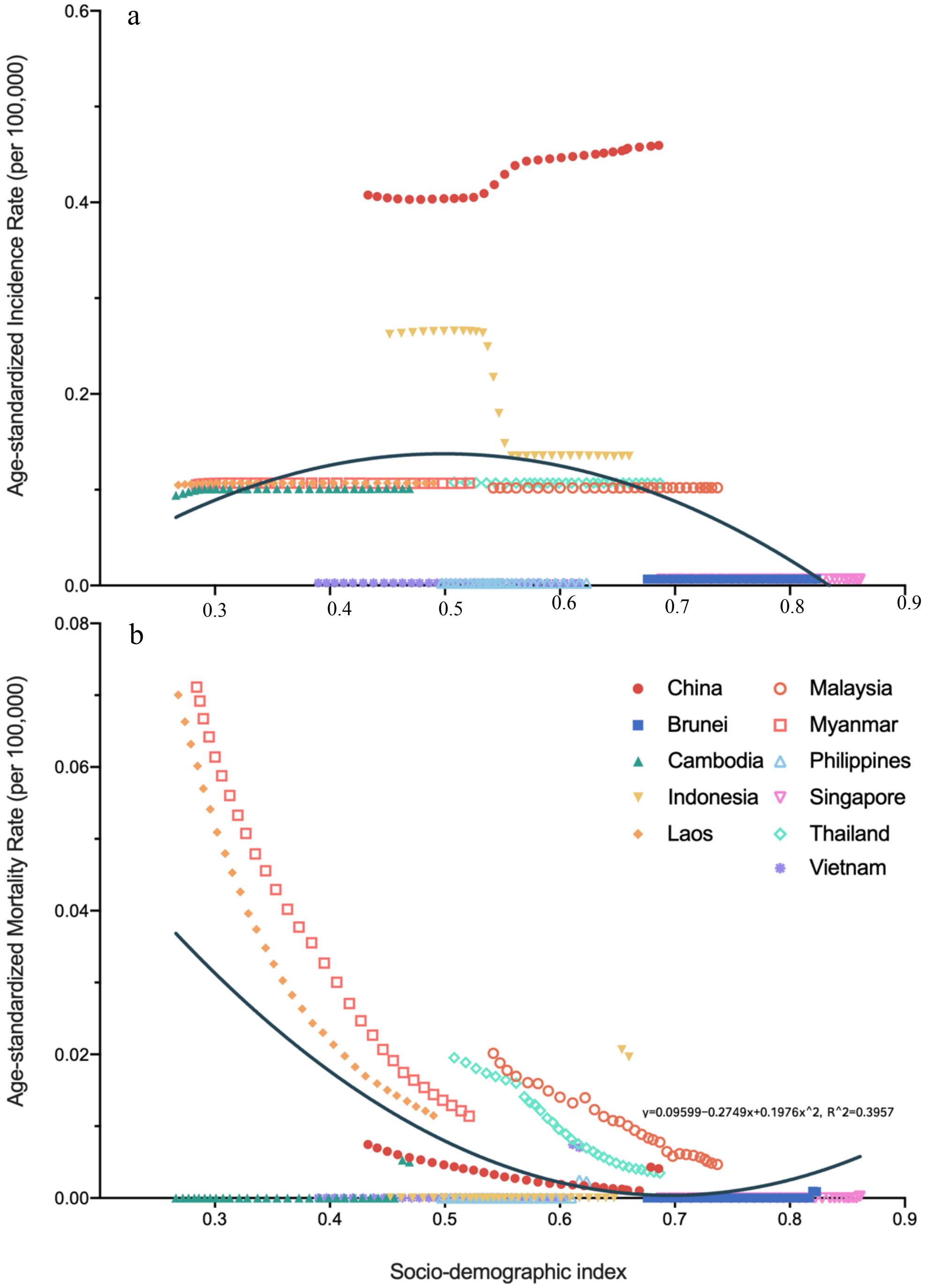

Supplementary Figure 5A. Cystic echinococcosis. a: association of Age-standardized incidence rate with Socio-demographic index; b: association of Age-standardized mortality rate with Socio-demographic index.

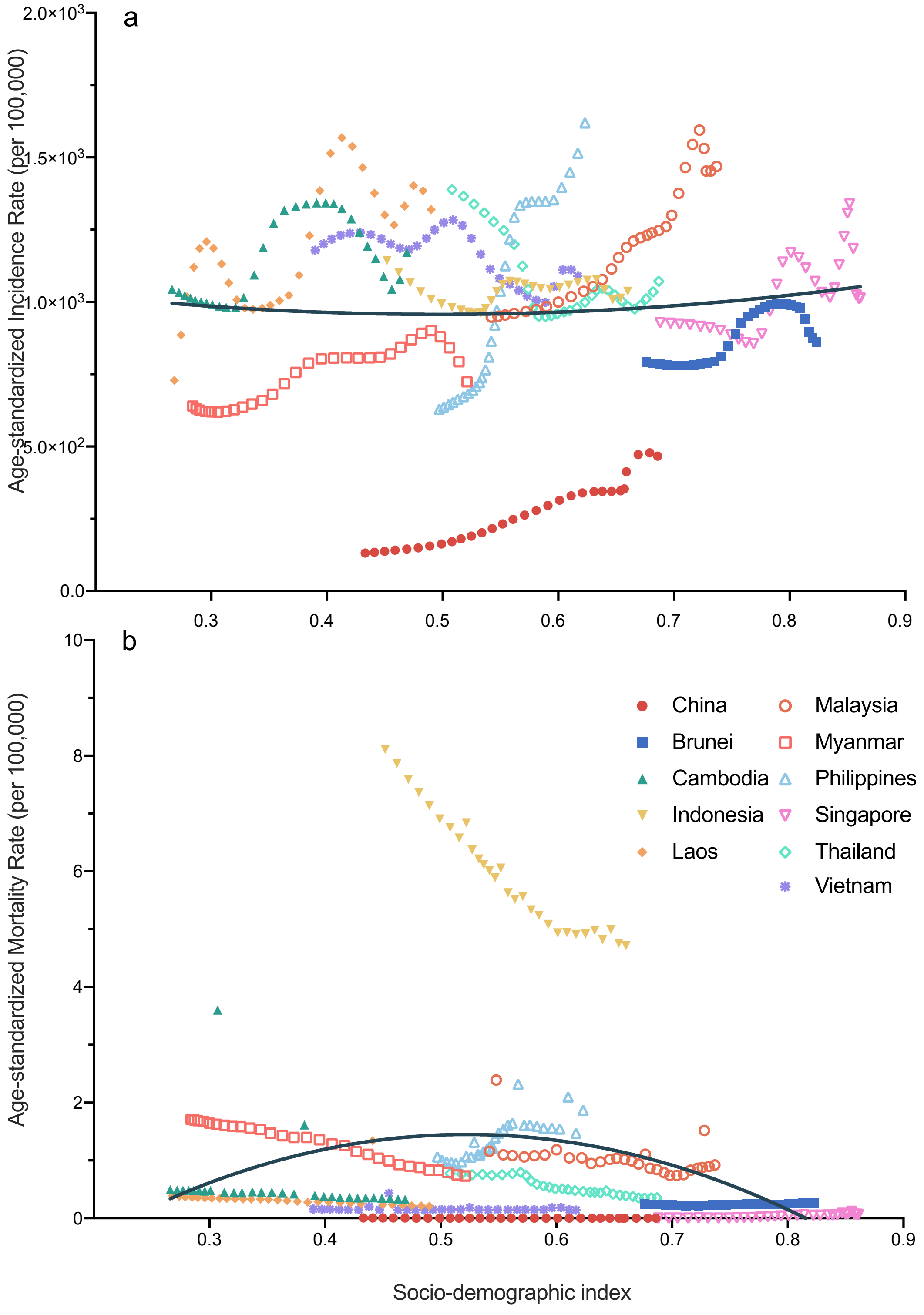

Supplementary Figure 5B. Dengue. a: association of Age-standardized incidence rate with Socio-demographic index; b: association of Age-standardized mortality rate with Socio-demographic index.

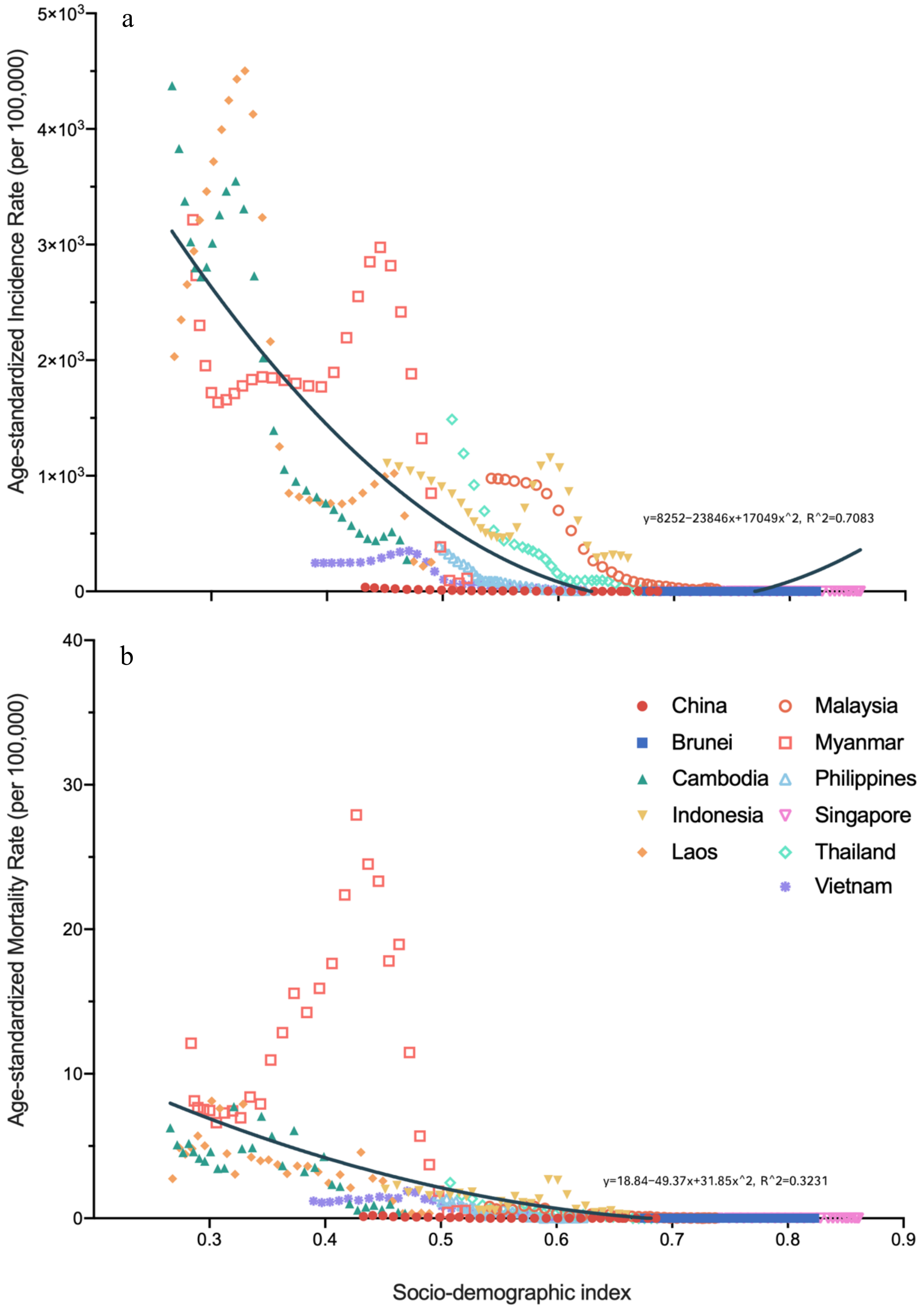

Supplementary Figure 5C. Malaria. a: association of Age-standardized incidence rate with Socio-demographic index; b: association of Age-standardized mortality rate with Socio-demographic index.

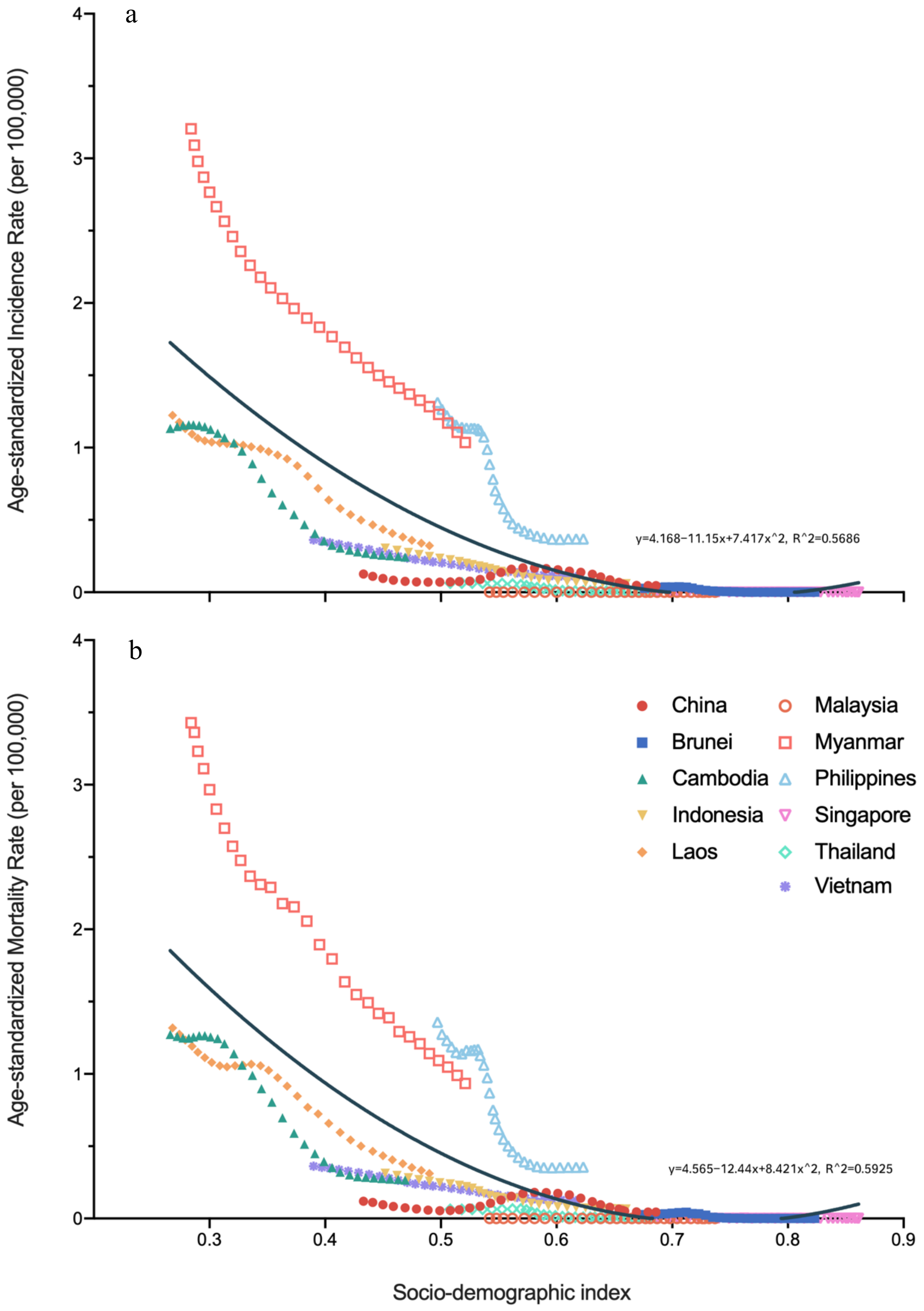

Supplementary Figure 5D. Rabies. a: association of Age-standardized incidence rate with Socio-demographic index; b: association of Age-standardized mortality rate with Socio-demographic index.
